# HAMAP as SPARQL rules—A portable annotation pipeline for genomes and proteomes

**DOI:** 10.1093/gigascience/giaa003

**Published:** 2020-02-08

**Authors:** Jerven Bolleman, Edouard de Castro, Delphine Baratin, Sebastien Gehant, Beatrice A Cuche, Andrea H Auchincloss, Elisabeth Coudert, Chantal Hulo, Patrick Masson, Ivo Pedruzzi, Catherine Rivoire, Ioannis Xenarios, Nicole Redaschi, Alan Bridge

**Affiliations:** 1 Swiss-Prot Group, SIB Swiss Institute of Bioinformatics, Centre Médical Universitaire, 1 rue Michel-Servet, CH-1211 Geneva 4, Switzerland; 2 Centre Hospitalier Universitaire Vaudois/Ludwig Institute for Cancer Research, Agora Centre, CH-1005 Lausanne, Switzerland

**Keywords:** protein, function, prediction, SPARQL

## Abstract

**Background:**

Genome and proteome annotation pipelines are generally custom built and not easily reusable by other groups. This leads to duplication of effort, increased costs, and suboptimal annotation quality. One way to address these issues is to encourage the adoption of annotation standards and technological solutions that enable the sharing of biological knowledge and tools for genome and proteome annotation.

**Results:**

Here we demonstrate one approach to generate portable genome and proteome annotation pipelines that users can run without recourse to custom software. This proof of concept uses our own rule-based annotation pipeline HAMAP, which provides functional annotation for protein sequences to the same depth and quality as UniProtKB/Swiss-Prot, and the World Wide Web Consortium (W3C) standards Resource Description Framework (RDF) and SPARQL (a recursive acronym for the SPARQL Protocol and RDF Query Language). We translate complex HAMAP rules into the W3C standard SPARQL 1.1 syntax, and then apply them to protein sequences in RDF format using freely available SPARQL engines. This approach supports the generation of annotation that is identical to that generated by our own in-house pipeline, using standard, off-the-shelf solutions, and is applicable to any genome or proteome annotation pipeline.

**Conclusions:**

HAMAP SPARQL rules are freely available for download from the HAMAP FTP site, ftp://ftp.expasy.org/databases/hamap/sparql/, under the CC-BY-ND 4.0 license. The annotations generated by the rules are under the CC-BY 4.0 license. A tutorial and supplementary code to use HAMAP as SPARQL are available on GitHub at https://github.com/sib-swiss/HAMAP-SPARQL, and general documentation about HAMAP can be found on the HAMAP website at https://hamap.expasy.org.

## Introduction

Continuing technological advances have reduced the costs of DNA sequencing enormously in recent years, leading to an explosion in the number of available whole-genome and metagenome sequences from all branches of the tree of life [1–5]. This wealth of sequence data presents exciting opportunities for experimental and computational research into the evolution and functional capacities of individual organisms and the communities they form, but fully exploiting these data will require complete and accurate functional annotation of these genome and metagenome sequences. Resources for genome annotation such as RAST/MG-RAST [6, 7], IMG/M [8], the NCBI genome annotation pipeline [9], InterPro [10], TIGRFAMS [11], and HAMAP [12] exploit information from experimentally characterized sequences to infer functions for uncharacterized homologs. While the underlying principles of these resources are undoubtedly similar, a lack of shared annotation standards and a suitable shared technical framework for annotation hamper efforts to use and combine them.

In this work, we use the HAMAP system to demonstrate technical solutions that could facilitate the combination and reuse of functional genome annotation systems from any provider. HAMAP classifies and annotates protein sequences using a collection of expert-curated protein family signatures and annotation rules. Swiss-Prot curators build HAMAP rules as part of an integrated workflow that includes curation of experimentally characterized template entries in UniProtKB/Swiss-Prot, as well as curation of the associated rule and protein family signature (encoded as a generalized profile). HAMAP rules annotate family members to the same level of detail and quality as the expert-curated UniProtKB/Swiss-Prot records on which they are based, combining family membership and residue dependencies to ensure a high degree of specificity [12].

The current implementation of HAMAP uses a custom rule format and annotation engine that are not easy to integrate into external pipelines. The HAMAP-Scan web service [13] is a good alternative for small research projects, but large genome-sequencing projects cannot depend on external web services to process large amounts of data. Our goal here was to develop a generic HAMAP rule format and annotation engine that is easily portable by external HAMAP users, using standard technologies that developers of other genome annotation pipelines could also adopt. To achieve this we have developed a representation of HAMAP annotation rules using the W3C standard SPARQL 1.1 syntax. SPARQL [14] is a query language for RDF [15], a core Semantic Web technology from the W3C. Our implementation allows users to apply HAMAP rules in SPARQL syntax to annotate protein sequences expressed as RDF using off-the-shelf SPARQL engines—without any need for a custom pipeline. If other annotation system providers were to adopt the same approach, it would then be possible to share and combine the annotation rules from multiple systems, execute them with any SPARQL engine, and compare the results.

## Methods

To use a generic SPARQL engine to execute rule-based protein sequence annotation, we need the following input data: (i) annotation rules in SPARQL syntax, (ii) protein sequence records in RDF syntax, and (iii) protein sequence/signature matches in RDF syntax, including alignment information for positional annotations.

**Figure 1:**
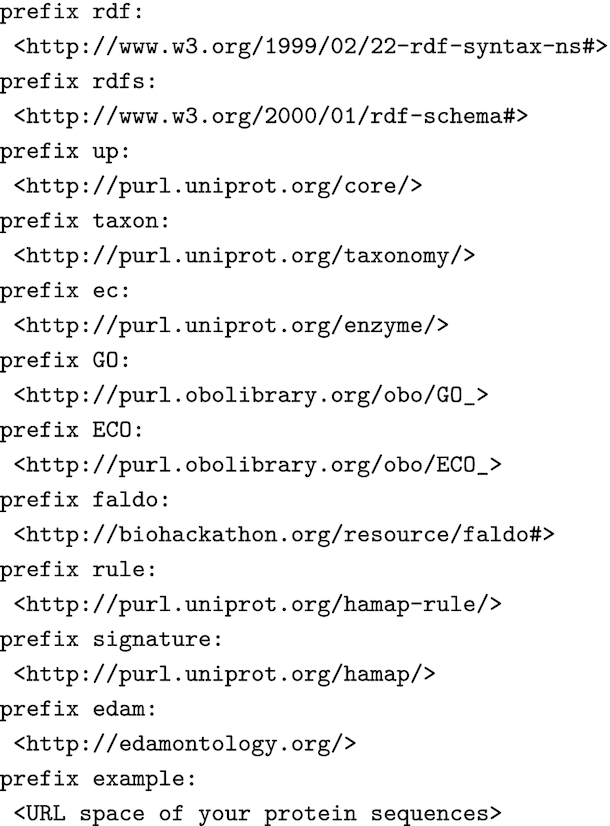
RDF namespace declarations for prefixes used in other figures.

To keep the examples given in the figures short, we provide all RDF namespace prefixes declarations in Fig. 1 and omit these from subsequent figures. We use the UniProt core ontology and other ontologies used by UniProt, such as the Feature Annotation Location Description Ontology (FALDO) [16], which is also used in the RDF of Ensembl [17] and Ensembl Genomes [18], to describe sequence positions, and the EDAM ontology [19] to describe sequence/signature matches.

### HAMAP annotation rules in SPARQL syntax

**Figure 2:**
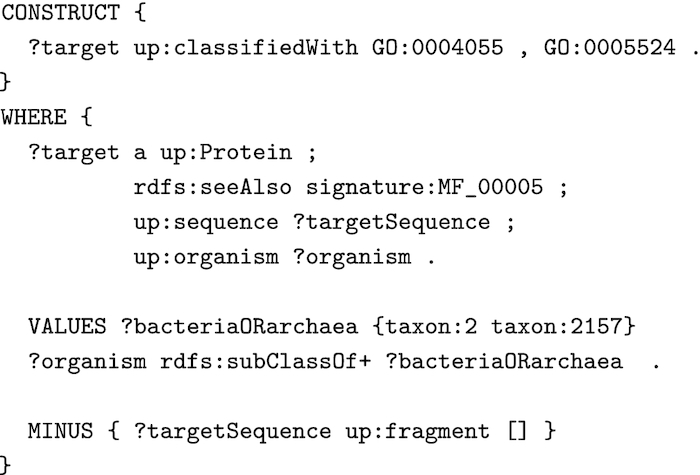
Part of the HAMAP rule for signature MF_00005 as a SPARQL CONSTRUCT query.

A HAMAP annotation rule consists of 2 parts: (i) the annotations and (ii) a set of conditions that must be satisfied in order to apply those annotations. The rule annotations can be expressed either by a CONSTRUCT block that returns the annotations as RDF triples or by an INSERT block that inserts these triples directly into an RDF store, while the rule conditions can be expressed by the WHERE clause of a SPARQL query. Fig. 2 shows part of the HAMAP rule for the signature MF_00005 as a SPARQL query. The CONSTRUCT block generates 2 annotations consisting of RDF triples for 2 Gene Ontology (GO) terms, providing that all conditions defined in the WHERE clause are satisfied. The conditions here are that the target must be a complete protein sequence, of bacterial or archaeal origin, and a member of the HAMAP family MF_00005 (i.e., matching the corresponding family signature).

**Figure 3:**
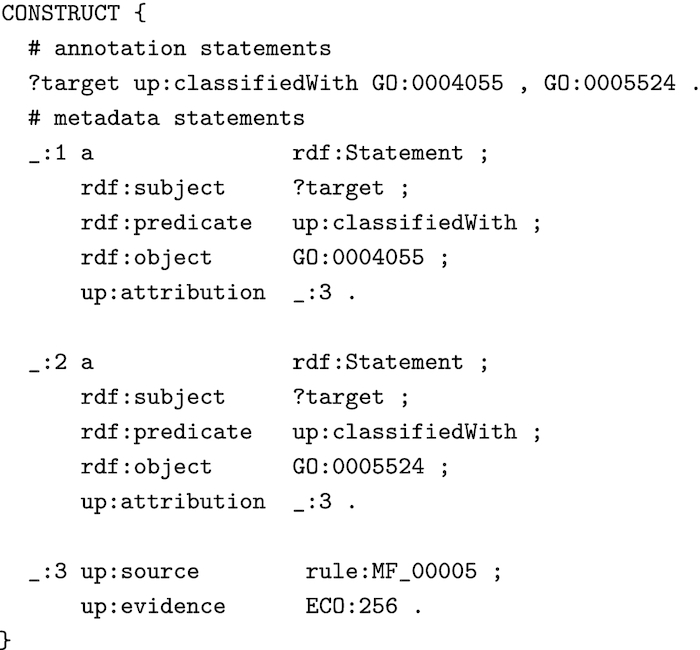
SPARQL CONSTRUCT block of Fig. 2 extended with metadata expressed as RDF reification quads.

Fig. 3 shows how the CONSTRUCT block of Fig. 2 can be extended to generate metadata for provenance and evidence for each annotation that the rule generates. We attribute the annotations to the HAMAP rule (MF_00005) and describe the type of the evidence with a value from the Evidence Code Ontology (ECO) [20]. We link the attribution to the annotations via RDF reification quads, which is verbose but is understood by all RDF syntaxes and data stores.

The original HAMAP rule implementation has 2 features that we have not yet implemented in this work. The first is the ability to call sequence analysis methods such as SignalP [21] and TMHMM [22] for the annotation of signal and transmembrane regions, which is not implemented here because these methods may not be available to external users. The second is precedence relationships between HAMAP rules, which are complex and apply to relatively few rules.

### Protein sequence records in RDF syntax

**Figure 4:**
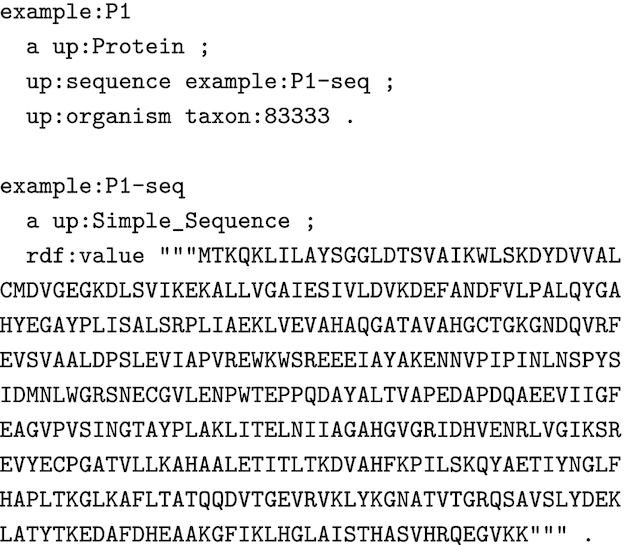
Example protein record in an RDF format suitable for HAMAP SPARQL rules.

HAMAP SPARQL rules require protein sequence records in a simple RDF format. Fig. 4 shows an example protein record with the identifier "P1" (example:P1). The rules require an identifier for the sequence (example:P1-seq) and the organism as an NCBI taxonomy identifier (taxon:83333). The actual protein sequence is provided as an IUPAC amino acid encoded string (in the rdf:value predicate of example:P1-seq) for positional annotations.

### Protein sequence/signature matches in RDF syntax

HAMAP SPARQL rules require sequence/signature match data in an RDF format.

Fig. 4 shows an RDF representation of the sequence/signature match of the example protein "P1" (Fig. 4) and the HAMAP signature MF_00005. The core information is a triple that states that the protein (example:P1) matches the signature (signature:MF_00005).

For positional annotations, the rule needs the start and end positions of the match region on the sequence, as well as the alignment between sequence and signature. We describe this information with the EDAM and FALDO ontologies and use the alignment format returned by the PfTools v3 [23] and InterProScan [10] software.

A HAMAP rule specifies the sequence positions of feature annotations—such as active sites or binding regions—with respect to 1 or more experimentally characterized "template" sequences in UniProtKB/Swiss-Prot. The rule engine therefore requires the alignment(s) of the template sequence(s) to the rule’s signature as input, and uses these to determine the corresponding positions on the template(s) and target sequence. A HAMAP rule may additionally require that the matching discrete position or range on the target sequence correspond to a specified amino acid or sequence motif, e.g., to check that an active site has the expected amino acid. This functionality can be implemented either in standard SPARQL 1.1 using the REPLACE, STRLEN, and CONCAT functions (see Supplementary Fig. S1 for an example), or via a custom SPARQL function (an example Java function for RDF stores that extends the Apache Jena ARQ SPARQL engine is given in Supplementary Fig. S2). We distribute the template sequence/signature alignments that are required for rule application together with the rules on our FTP site [24].

### Simplifying the output from HAMAP rules for other annotation pipelines

HAMAP rules provide functional annotation in the form of free text and using controlled vocabularies and ontologies developed by UniProt and others. These include GO [25], the Enzyme Classification of the IUBMB (“EC numbers”) [26] represented by the ENZYME database [27], and the Rhea database of biochemical reactions [28] based on the ChEBI ontology [29]. Each HAMAP rule provides all annotation fields required in UniProtKB. For users requiring only a subset of these annotations—such as enzymatic reactions described using Rhea, or protein functions, processes, and cellular components described using GO—it is possible to translate only the desired annotation types into SPARQL queries. We can also modify the CONSTRUCT/INSERT block of the queries to return the results as simple protein-annotation associations (see Table 1). This tabular result format can easily be loaded into a relational database or JSON-based document store and requires no further investment in a Semantic Web technology stack.

**Table 1:** Simple protein-annotation associations of HAMAP rule MF_00005 for UniProtKB entry B1YJ35

Protein	Annotation
uniprot:B1YJ35	“GO:0004055”
uniprot:B1YJ35	“GO:0005524”
uniprot:B1YJ35	“GO:0006526”
uniprot:B1YJ35	“GO:0005737”
uniprot:B1YJ35	“ec:6.3.4.5”
uniprot:B1YJ35	“rhea:10932”

## Results

### Validation

**Figure 5:**
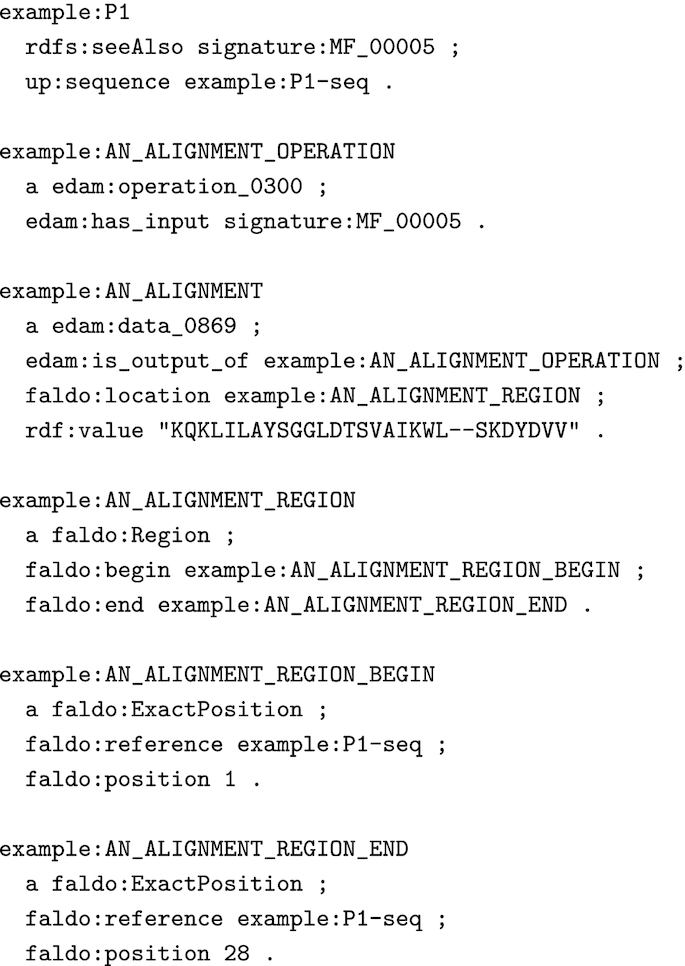
Example protein sequence/signature match in RDF syntax.

We have tested the approach of executing rule-based annotation with a generic SPARQL engine with the data from the HAMAP and UniProtKB/Swiss-Prot releases 2019_10. We translated the HAMAP rules into SPARQL CONSTRUCT queries and the protein sequences into the RDF format described in Fig. 4. We generated the RDF representation of the sequence/signature matches, as illustrated in Fig. 5, directly from a relational database containing the results of PfTools v3.1 scans of UniProtKB/Swiss-Prot versus HAMAP for our internal HAMAP release pipeline. Other groups could achieve the same result by scanning their protein sequences with PfTools v3.2 [30], which has a new output option for RDF format, or with InterProScan and converting the XML result files into RDF format with the XSLT stylesheet that we provide for this purpose [31].

**Figure 6:**
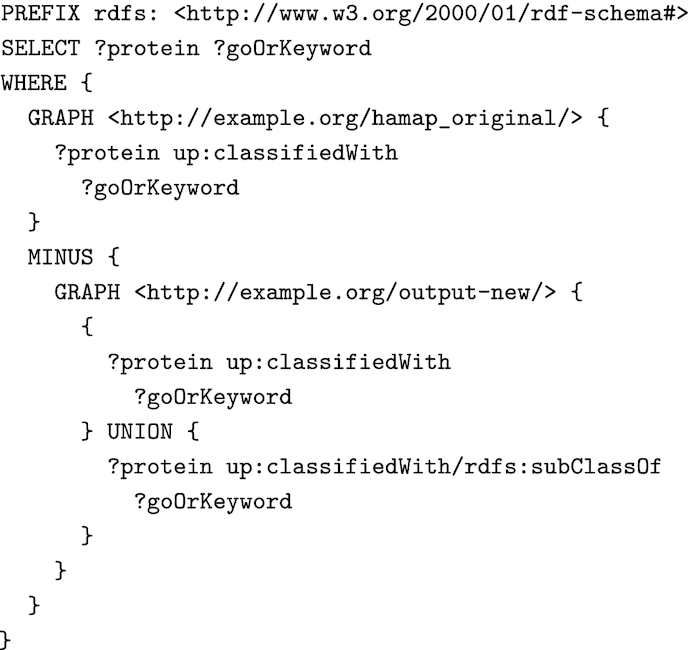
Example query for comparison of annotations generated by the different systems, taking into account whether a system inserts the full GO or UniProt keyword hierarchy or only leaf nodes.

We tested 2 different open-source SPARQL engines (Virtuoso RDF 7.2 and Apache Jena TDB2 3.13.1) to execute our rules and validated the generated annotations by comparing them to those obtained from our custom platform. This platform, implemented in Scala/Java, uses as input files protein entries in FASTA format and HAMAP rules in their custom text format to generate annotations in UniProtKB format (text, XML, or RDF). The RDF data generated by the different systems was loaded into separate named graphs of an RDF database for comparisons using SPARQL queries to search for annotations unique to any of the 3 runs (see example query in Fig. 6). The existing custom HAMAP annotation pipeline and each of the 2 SPARQL engines generated identical annotations, except for those that depend on external sequence analysis methods and the evaluation of HAMAP rule precedence, which we did not implement here as described in section HAMAP annotation rules in SPARQL syntax.

On a laptop with 8 cores, it takes ∼4 minutes to scan a small *Escherichia coli* proteome with the HAMAP signatures using PfTools v3.2 or InterProScan software, and 1 minute to execute the HAMAP rules with Apache Jena TDB2 (see the instructions in the tutorial [32]). This shows that the sequence/signature scanning step is the bottleneck in our system. Both steps, scanning and rule execution, could be run in an embarrassingly parallel fashion. A further optimization for high-performance computing would be to avoid HTTP communication by running the SPARQL query reader and processor in the same process.

An additional small benefit of the SPARQL representation is that SPARQL queries can be serialized in RDF and loaded into a SPARQL engine. We set up a server with our rules [33] that allows us to perform quality assurance on our rules by running analytical queries across them and SPARQL endpoints of other life science resources.

## Discussion

### Protein function annotation pipelines based on SPARQL

Here we have developed a SPARQL representation of HAMAP annotation rules that allows other groups with basic knowledge of this widespread standard technology to incorporate HAMAP in their own genome and proteome annotation pipelines. SPARQL can express all features of complex HAMAP rules, including the logic required for positional annotations, while freely available SPARQL engines provide a means to execute HAMAP rules without recourse to specialized software. This work demonstrates the feasibility of adopting SPARQL as a means to integrate existing functional annotation pipelines for genome-sequencing projects. This applies not only to expert curated rules from HAMAP and other systems but also annotation rules generated by automated approaches such as deep learning [34,35], which require a feature vector to be expressed as an RDF triple as shown by Linked Open Data for Machine Learning (LOD4ML) [36]. SPARQL can also be adopted by those without access to specialized RDF triple stores by using a SPARQL to SQL mapping (such as that provided by any of the R2RML tools [37]) to execute SPARQL rules directly against data stored in a relational database. The main weakness of SPARQL is that, like many generic query engines, it tends to be computationally more expensive than a custom solution, but we have seen significant progress in the optimization of SPARQL engines in recent years [38].

### An approach that is extensible to any domain of biology

**Figure 7:**
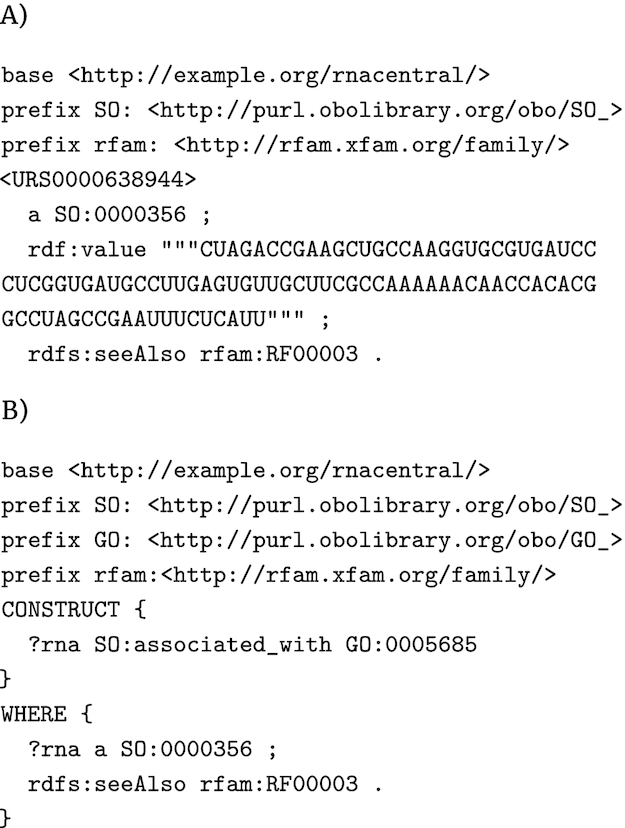
(A) Hypothetical triples to describe a sequence entry from RNAcentral.org that is a member of the Rfam RNA family RF00003 (U1 spliceosomal RNA family). (B) Hypothetical rule associating RF00003 to the GO term GO:0005685 (definition: “A ribonucleoprotein complex that contains small nuclear RNA U1”).

While we have limited our demonstration to the use of SPARQL queries to formalize and execute protein annotation rules from HAMAP, there is nothing that ties the SPARQL approach to a particular domain of biology. Complete genome annotation requires identification and functional annotation of RNAs as well as proteins, and Fig. 7 provides a demonstration of how that annotation could be provided by SPARQL. Here a hypothetical SPARQL rule specifies functional (GO) annotation for an RNA sequence of RNAcentral [39] that is a member of the U1 spliceosomal RNA family as defined by Rfam [40].

The development of annotation rules for a given domain across different groups will require community standards for the representation of the relevant domain-specific annotation types. In this work we have used the RDF vocabularies of UniProt, which allowed us to easily compare the results of the SPARQL approach to those of our existing HAMAP rule annotation pipeline. As other appropriate community ontologies become available, our queries and SPARQL rules can be easily adapted.

### Further work

We plan to further extend our implementation of HAMAP rules using SPARQL to include external method calls and deal with rule precedence (see Section HAMAP annotation rules in SPARQL syntax), and also develop a SPARQL representation for PROSITE, which provides protein domain annotation via a custom pipeline, ScanProsite [41]. HAMAP and PROSITE are 2 of the main components of the UniRule system of UniProt, which provides automatic annotation for unreviewed entries of UniProtKB/TrEMBL [42], and the approach described here could be extended to the entire UniRule system. The UniProt data model was recently extended to allow enzyme annotation using biochemical reaction data from the Rhea database [43], which will further extend the scope of HAMAP SPARQL rules to more specialized applications—such as the creation and annotation of draft networks of metabolic reactions [44,45].

## Availability of Supporting Source Code and Requirements

- Project name: HAMAP
- Project home page: https://hamap.expasy.org
- Other requirements: SPARQL 1.1–compliant RDF store, sequence/signature scanning software (e.g., PfTools v3.2, biotools:pfsearch, or InterProScan, RRID:SCR_005829)
- License: CC-BY-ND 4.0
- RRID:SCR_007701

## Availability of Supporting Data and Materials

The data sets supporting the results of this article are available in the GigaDB repository [46].

## Additional Files

**Supplementary Information S1**. Map position on template to target using SPARQL 1.1. standard functions.

**Supplementary Information S2**. Java Apache Jena ARQ Custom Function.

**Supplementary Information S3**. XSLT to covert InterProScan XML output to minimal RDF for HAMAP.

giaa003_GIGA-D-19-00242_Original_Submission

giaa003_GIGA-D-19-00242_Revision_1

giaa003_GIGA-D-19-00242_Revision_2

giaa003_Response_to_Reviewer_Comments_Original_Submission

giaa003_Response_to_Reviewer_Comments_Revision_1

giaa003_Reviewer_1_Report_Original_Submission Mark Wass -- 8/12/2019 Reviewed

giaa003_Reviewer_2_Report_Original_Submission Weizhong Li -- 8/30/2019 Reviewed

giaa003_Supplemental_File

## Abbreviations

ECO: Evidence Code Ontology; FALDO: Feature Annotation Location Description Ontology; GO: Gene Ontology; HAMAP: High-quality Automated and Manual Annotation of Proteins; IUPAC: International Union of Pure and Applied Chemistry; JSON: JavaScript Object Notation; LOD4ML: Linked Open Data for Machine Learning; NCBI: National Center for Biotechnology Information; RDF: Resource Description Framework; SIB: Swiss Institute of Bioinformatics; SPARQL: SPARQL Protocol and RDF Query Language; W3C: World Wide Web Consortium.

## Competing interests

The authors declare that they have no competing interests.

## Funding

HAMAP activities at the SIB are supported by the Swiss Federal Government through the State Secretariat for Education, Research and Innovation SERI, and the Swiss National Science Foundation (SNSF). The development of the HAMAP SPARQL rules was also supported by the ELIXIR Implementation study on “A microbial metabolism resource for Systems Biology.” Funding for open access charge: SERI.

## Authors' contributions

J.B. designed the HAMAP-SPARQL system, implemented the software to convert HAMAP rules to SPARQL, ran the analysis, and co-wrote the manuscript. E.d.C. implemented the original HAMAP pipeline and assisted with the translation of the rules to SPARQL. D.B. implemented software to extract sequence/signature matches in RDF format from an Oracle database. S.G. helped to optimize SPARQL queries and define the rule data model. B.A.C. helped to define the RDF format for sequence/signature matches. A.H.A., E.C., C.H., P.M., I.P., and C.R. curate Swiss-Prot entries and HAMAP rules used in this manuscript. I.X. participated in the planning of the project. N.R. tested and revised the tutorial and co-wrote the manuscript. A.B. co-wrote and edited the manuscript. All authors provided critical feedback on the project.
