# Supplementary material for: HAMAP as SPARQL rules—A portable annotation pipeline for genomes and proteomes
Source: Gigascience. 2020 Feb 8;9(2):giaa003. doi: 10.1093/gigascience/giaa003 (PMC7007698; doi:10.1093/gigascience/giaa003)
Supplement: giaa003_GIGA-D-19-00242_Revision_1 [file giaa003_giga-d-19-00242_revision_1.pdf]

GigaScience

HAMAP as SPARQL rules – A portable annotation pipeline for genomes and proteomes

--Manuscript Draft--

**Manuscript Number:**	GIGA-D-19-00242R1
**Full Title:**	HAMAP as SPARQL rules – A portable annotation pipeline for genomes and proteomes
**Article Type:**	Research
**Funding Information:**	Staatssekretariat für Bildung, Forschung und Innovation (CH) — Dr Alan Bridge
**Abstract:**	Genome and proteome annotation pipelines are generally custom built and therefore not easily reusable by other groups, which leads to duplication of effort, increased costs, and suboptimal results. One cost-effective way to increase the data quality in public databases is to encourage the adoption of annotation standards and technological solutions that enable the sharing of biological knowledge and tools for genome and proteome annotation. We have translated the rules of our HAMAP proteome annotation pipeline to queries in the W3C standard SPARQL 1.1 syntax and applied them with two off-the-shelf SPARQL engines to UniProtKB/Swiss-Prot protein sequences described in RDF format. This approach is applicable to any genome or proteome annotation pipeline and greatly simplifies their reuse. HAMAP SPARQL rules and documentation are freely available for download from the HAMAP FTP site ftp://ftp.expasy.org/databases/hamap/hamap_sparql.tar.gz under a CC-BY-ND 4.0 license. The annotations generated by the rules are under the CC-BY 4.0 license.
**Corresponding Author:**	Jerven Tjalling Bolleman<br>Swiss Institute of Bioinformatics Geneva<br>Geneva, GE SWITZERLAND
**Corresponding Author Secondary Information:**	
**Corresponding Author's Institution:**	Swiss Institute of Bioinformatics Geneva
**Corresponding Author's Secondary Institution:**	
**First Author:**	Jerven Tjalling Bolleman
**First Author Secondary Information:**	
**Order of Authors:**	Jerven Tjalling Bolleman
	Eduoard de Castro
	Delphine Baratin
	Sebastien Gehant
	Beatrice A Cuche
	Andrea H Auchincloss
	Elisabeth Coudert
	Chantal Hulo
	Patrick Masson
	Ivo Pedruzzi
	Catherine Rivoire
	Ioannis Xenarios
	Nicole Redaschi

Powered by Editorial Manager® and ProduXion Manager® from Aries Systems Corporation

	Alan Bridge
**Order of Authors Secondary Information:**	
**Response to Reviewers:**	Dear Reviewers, Curators, Editor,<br><br>Thank you for your kind reviews and patience.<br><br>===========<br><br>COMMENT: a key requirement for GigaScience is usability, and more detailed documentation, tutorial and example should be provided.<br>ANSWER: We have published documentation and a tutorial at https://github.com/sib-swiss/HAMAP-SPARQL<br><br>COMMENT: please register any new software application in the bio.tools and SciCrunch.org databases to receive RRID (Research Resource Identification Initiative ID) and biotoolsID identifiers, and include these in your manuscript. This will facilitate tracking, reproducibility and re-use of your tool.<br><br>ANSWER: HAMAP is already registered with RRID:SCR_007701.<br><br>Reviewer #1:<br>============<br><br>COMMENT: No citation or other information is provided to support this claim on the level of detail and quality of the annotations that HAMAP rules produce. Either a citation to work showing this should be provided or the sentence altered.<br><br>ANSWER: The process of UniProtKB/Swiss-Prot curation and HAMAP rule curation are tightly coupled. HAMAP rules are created by the same Swiss-Prot curators that curate the template entries. These curators then extract the relevant annotations into HAMAP rules. The HAMAP rules are also checked against the content of matching UniProtKB/Swiss-Prot entries at each release - to ensure that the rules remain consistent with the current contents of UniProtKB/Swiss-Prot. So we can reasonably say that the HAMAP system is of the same detail and quality as UniProtKB/Swiss-Prot. We have added the missing citation that describes this in more detail. Interested users can search for HAMAP annotation at uniprot.org using this url: https://www.uniprot.org/uniprot/?query=reviewed:yes+source:hamap.<br><br>Reviewer #2:<br>============<br><br>COMMENT: The package (ftp://ftp.expasy.org/databases/hamap/hamap_sparql.tar.gz) only contains several data files, without documentation<br>ANSWER: We have added a README file to the package that describes where to find the documentation. This points to https://github.com/sib-swiss/HAMAP-SPARQL.<br><br>COMMENT: It will be more convincing if rules from another popular annotation pipeline cited in the paper can be made into SPARQL query. This can be included either in the paper, supplementary materials, or an example packed with the software.<br><br>ANSWER: One of the most popular annotation tools is InterPro2GO (described in the already cited InterPro paper). We show how to translate these simple association rules in https://github.com/sib-swiss/HAMAP-SPARQL/blob/master/INTERPRO2GO.md.<br><br>COMMENT: Supplementary document is in Latex format, which is not very convenient to read. I suggest convert it to PDF format.<br>ANSWER: Our apologies, we had expected the gigascience pipeline to

	have done this for us, We have also published the code contained in<br>the supplement at<br>https://github.com/sib-swiss/HAMAP-SPARQL<br><br>COMMENT: Documentation of the tool is very limited. More detailed<br>documentation, tutorial and example should be provided.<br>ANSWER: We have published documentation and a tutorial at<br>https://github.com/sib-swiss/HAMAP-SPARQL<br><br>COMMENT: Citations were not correctly ordered.<br>ANSWER: We have reordered the citations according to the guidelines for authors.<br><br>Curators:<br>============<br><br>To make it possible to reproduce the query described in Figure 6 we<br>have the data required in nquads format in a compressed form. We would<br>like to upload this to gigadb.<br><br><br>ALL:<br>===========<br><br>We also rephrased the section on performance to apply to a more common<br>to understand task, annotating a bacterial genome instead of a whole<br>Swiss-Prot release.<br><br>With sincere regards,<br>The authors
**Additional Information:**	
**Question**	**Response**
Are you submitting this manuscript to a special series or article collection?	No
**Experimental design and statistics**<br><br>Full details of the experimental design and statistical methods used should be given in the Methods section, as detailed in our Minimum Standards Reporting Checklist. Information essential to interpreting the data presented should be made available in the figure legends.<br><br>Have you included all the information requested in your manuscript?	No
If not, please give reasons for any omissions below.<br><br>as follow-up to "**Experimental design and statistics**	Not applicable

Full details of the experimental design and statistical methods used should be given in the Methods section, as detailed in our Minimum Standards Reporting Checklist. Information essential to interpreting the data presented should be made available in the figure legends.<br><br>Have you included all the information requested in your manuscript?<br><br>"	
**Resources**<br><br>A description of all resources used, including antibodies, cell lines, animals and software tools, with enough information to allow them to be uniquely identified, should be included in the Methods section. Authors are strongly encouraged to cite Research Resource Identifiers (RRIDs) for antibodies, model organisms and tools, where possible.<br><br>Have you included the information requested as detailed in our Minimum Standards Reporting Checklist?	Yes
**Availability of data and materials**<br><br>All datasets and code on which the conclusions of the paper rely must be either included in your submission or deposited in publicly available repositories (where available and ethically appropriate), referencing such data using a unique identifier in the references and in the "Availability of Data and Materials" section of your manuscript.<br><br>Have you have met the above requirement as detailed in our Minimum Standards Reporting Checklist?	Yes

LaTeX - Main Document (TeX file)

Click here to access/download;LaTeX - Main Document (TeX file);bolleman.tex

Placeholder for OUP logo oup.pdf

Placeholder for journal logo gigascience-logo.pdf

GigaScience, 2017, 1–6

doi: xx.xxxx/xxxx
Manuscript in Preparation
Paper

PAPER

HAMAP as SPARQL rules – A portable annotation pipeline for genomes and proteomes

Jerven Bolleman[1,*], Edouard de Castro[1], Delphine Baratin[1], Sebastien Gehant[1], Beatrice A. Cuche[1], Andrea H. Auchincloss[1], Elisabeth Coudert[1], Chantal Hulo[1], Patrick Masson[1], Ivo Pedruzzi[1], Catherine Rivoire[1], Ioannis Xenarios[1,2], Nicole Redaschi[1] and and Alan Bridge[1]

[1]Swiss-Prot Group, SIB Swiss Institute of Bioinformatics, CMU, 1 rue Michel-Servet, CH-1211 Geneva 4, Switzerland and [2]CHUV/LICR, Agora Centre, CH-1005 Lausanne, Switzerland

*jerven.bolleman@sib.swiss, hamap@sib.swiss

Abstract

Genome and proteome annotation pipelines are generally custom built and not easily reusable by other groups. This leads to duplication of effort, increased costs, and suboptimal annotation quality. One way to address these issues is to encourage the adoption of annotation standards and technological solutions that enable the sharing of biological knowledge and tools for genome and proteome annotation.
Here we demonstrate one approach to generate portable genome and proteome annotation pipelines that users can run without recourse to custom software. This proof of concept uses our own rule-based annotation pipeline HAMAP, which provides functional annotation for protein sequences to the same depth and quality as UniProtKB/Swiss-Prot, and the W3C standards RDF and SPARQL. We translate complex HAMAP rules into the W3C standard SPARQL 1.1 syntax, and then apply them to protein sequences in RDF format using freely available SPARQL engines. This approach supports the generation of annotation that is identical to that generated by our own in-house pipeline, using standard, off the shelf solutions, and is applicable to any genome or proteome annotation pipeline.
HAMAP SPARQL rules and documentation are freely available for download from the HAMAP FTP site ftp://ftp.expasy.org/databases/hamap/hamap_sparql.tar.gz under a CC-BY-ND 4.0 license. The annotations generated by the rules are under the CC-BY 4.0 license.
A tutorial and supplementary code to use HAMAP as SPARQL is provided at https://github.com/sib-swiss/HAMAP-SPARQL.

Key words: Protein, Function, Prediction, SPARQL

Introduction

Continuing technological advances have reduced the costs of DNA sequencing enormously in recent years, leading to an explosion in the number of available whole genome and metagenome sequences from all branches of the tree of life [1, 2, 3, 4, 5]. This wealth of sequence data presents exciting opportunities for experimental and computational research into the evolution and functional capacities of individual organisms and the communities they form, but fully exploiting this data will require complete and accurate functional annotation of these genome and metagenome sequences. Resources for genome annotation such as RAST/MG-RAST [6, 7], IMG/M [8], the NCBI genome annotation pipeline [9], InterPro [10], TIGRFAMS [11], and HAMAP [12] exploit information from experimentally characterized sequences to infer functions for uncharacterized homologs. While the underlying principles of these resources are undoubtedly very similar, a lack of shared annotation standards and a suitable shared technical framework for annotation hamper efforts to use and combine them.

In this work, we use the HAMAP system (https://hamap.expasy.org) to demonstrate technical solutions that could facilitate the combination and reuse of functional genome annotation systems from any provider. HAMAP classifies and annotates protein sequences using a collection of expert-curated protein family signatures and annotation rules. Swiss-Prot curators build HAMAP rules as part of an integrated workflow that includes curation of experimentally characterized template entries in UniProtKB/Swiss-Prot, as well as curation of the associated rule and protein family signature (encoded as a generalized profile). HAMAP rules annotate family members to the same level of detail and quality as the expert curated UniProtKB/Swiss-Prot records

on which they are based, combining family membership and residue dependencies to ensure a high degree of specificity [12].

The current implementation of HAMAP uses a custom rule format and annotation engine that are not easy to integrate into external pipelines. The HAMAP-Scan web service (https://hamap.expasy.org/hamap_scan.html) is a good alternative for small research projects, but large genome sequencing projects cannot depend on external web services to process large amounts of data. Our goal here was to develop a generic HAMAP rule format and annotation engine that is easily portable by external HAMAP users, using standard technologies that developers of other genome annotation pipelines could also adopt. To achieve this we have developed a representation of HAMAP annotation rules using the World Wide Web Consortium (W3C) standard SPARQL 1.1 syntax. SPARQL (a recursive acronym for the SPARQL Protocol and RDF Query Language) is a query language for RDF (Resource Description Framework), a core Semantic Web technology from the W3C (see https://www.w3.org/RDF/ for more details). Our implementation allows users to apply HAMAP rules in SPARQL syntax to annotate protein sequences expressed as RDF using off-the-shelf SPARQL engines – without any need for a custom pipeline. If other annotation system providers were to adopt the same approach, it would then be possible to share and combine the annotation rules from multiple systems, execute them with any SPARQL engine, and compare the results.

Methods

To use a generic SPARQL engine to execute rule-based protein sequence annotation, we need the following input data: a) annotation rules in SPARQL syntax, b) protein sequence records in RDF syntax, and c) protein sequence/signature matches in RDF syntax, including alignment information for positional annotations.

To keep the examples given in the Figures short, we provide all RDF namespace prefixes declarations in Figure 1 and omit these from subsequent Figures. We use the UniProt core ontology and other ontologies used by UniProt, such as FALDO [13], which is also used in the RDF of Ensembl [14] and Ensembl Genomes [15], to describe sequence positions, and the EDAM ontology [16] to describe sequence/signature matches.

HAMAP annotation rules in SPARQL syntax

A HAMAP annotation rule consists of two parts: 1) the annotations, and 2) a set of conditions that must be satisfied in order to apply those annotations. The rule annotations can be expressed either by a CONSTRUCT block that returns the annotations as RDF triples or by an INSERT block that inserts these triples directly into an RDF store, while the rule conditions can be expressed by the WHERE clause of a SPARQL query. Figure 2 shows part of the HAMAP rule for the signature MF_00005 as a SPARQL query. The CONSTRUCT block generates two annotations consisting of RDF triples for two Gene Ontology (GO) terms, providing that all conditions defined in the WHERE clause are satisfied. The conditions here are that the target must be a complete protein sequence, of bacterial or archaeal origin, and a member of the HAMAP family MF_00005 (i.e. matching the corresponding family signature).

Figure 3 shows how the CONSTRUCT block of Figure 2 can be extended to generate metadata for provenance and evidence for each annotation that the rule generates. We attribute the annotations to the HAMAP rule (MF_00005) and describe the type of the evidence with a value from the Evidence Code On-

```
prefix rdf:
 <http://www.w3.org/1999/02/22-rdf-syntax-ns#>
prefix rdfs:
 <http://www.w3.org/2000/01/rdf-schema#>
prefix up:
 <http://purl.uniprot.org/core/>
prefix taxon:
 <http://purl.uniprot.org/taxonomy/>
prefix ec:
 <http://purl.uniprot.org/enzyme/>
prefix GO:
 <http://purl.obolibrary.org/obo/GO_>
prefix ECO:
 <http://purl.obolibrary.org/obo/ECO_>
prefix faldo:
 <http://biohackathon.org/resource/faldo#>
prefix rule:
 <http://purl.uniprot.org/hamap-rule/>
prefix signature:
 <http://purl.uniprot.org/hamap/>
prefix edam:
 <http://edamontology.org/>
prefix example:
 <URL space of your protein sequences>
```

Figure 1. RDF namespace declarations for prefixes used in other Figures.

```
CONSTRUCT {
  ?target up:classifiedWith GO:0004055 , GO:0005524 .
}
WHERE {
  ?target a up:Protein ;
          rdfs:seeAlso signature:MF_00005 ;
          up:sequence ?targetSequence ;
          up:organism ?organism .

  VALUES ?bacteriaORarchaea {taxon:2 taxon:2157}
  ?organism rdfs:subClassOf+ ?bacteriaORarchaea  .

  MINUS { ?targetSequence up:fragment [] }
}
```

Figure 2. Part of the HAMAP rule for signature MF_00005 as a SPARQL CONSTRUCT query.

```
CONSTRUCT {
  # annotation statements
  ?target up:classifiedWith GO:0004055 , GO:0005524 .
  # metadata statements
  _:1 a               rdf:Statement ;
      rdf:subject     ?target ;
      rdf:predicate   up:classifiedWith ;
      rdf:object      GO:0004055 ;
      up:attribution  _:3 .

  _:2 a               rdf:Statement ;
      rdf:subject     ?target ;
      rdf:predicate   up:classifiedWith ;
      rdf:object      GO:0005524 ;
      up:attribution  _:3 .

  _:3 up:source        rule:MF_00005 ;
      up:evidence      ECO:256 .
}
```

Figure 3. SPARQL CONSTRUCT block of Fig. 2 extended with metadata expressed as RDF reification quads.

```
example:P1
  a up:Protein ;
  up:sequence example:P1-seq ;
  up:organism taxon:83333 .

example:P1-seq
  a up:Simple_Sequence ;
  rdf:value """MTKQKLILAYSGGLDTSVAIKWLSKDYDVVAL
CMDVGEGKDLSVIKEKALLVGAIESIVLDVKDEFANDFVLPALQYGA
HYEGAYPLISALSRPLIAEKLVEVAHAQGATAVAHGCTGKGNDQVRF
EVSVAALDPSLEVIAPVREWKWSREEEIAYAKENNVPIPINLNSPYS
IDMNLWGRSNECGVLENPWTEPPQDAYALTVAPEDAPDQAEEVIIGF
EAGVPVSINGTAYPLAKLITELNIIAGAHGVGRIDHVENRLVGIKSR
EVYECPGATVLLKAHAALETITLTKDVAHFKPILSKQYAETIYNGLF
HAPLTKGLKAFLTATQQDVTGEVRVKLYKGNATVTGRQSAVSLYDEK
LATYTKEDAFDHEAAKGFIKLHGLAISTHASVHRQEGVKK""" .
```

Figure 4. Example protein record in an RDF format suitable for HAMAP SPARQL rules.

tology (ECO) [17]. We link the attribution to the annotations via RDF reification quads, which is verbose but is understood by all RDF syntaxes and data stores.

The original HAMAP rule implementation has two features that we have not yet implemented in this work. The first is the ability to call sequence analysis methods such as SignalP [18] and TMHMM [19] for the annotation of signal and transmembrane regions, which is not implemented here as these methods may not be available to external users. The second is precedence relationships between HAMAP rules, which are complex and apply to relatively few rules.

Protein sequence records in RDF syntax

HAMAP SPARQL rules require protein sequence records in a simple RDF format. Figure 4 shows an example protein record with the identifier ‘P1’ (example:P1). The rules require an identifier for the sequence (example:P1-seq) and the organism as an NCBI taxonomy identifier (taxon:83333). The actual protein sequence is provided as an IUPAC amino acid encoded string (in the rdf:value predicate of example:P1-seq) for positional annotations.

```
example:P1
  rdfs:seeAlso signature:MF_00005 ;
  up:sequence example:P1-seq .

example:AN_ALIGNMENT_OPERATION
  a edam:operation_0300 ;
  edam:has_input signature:MF_00005 .

example:AN_ALIGNMENT
  a edam:data_0869 ;
  edam:is_output_of example:AN_ALIGNMENT_OPERATION ;
  faldo:location example:AN_ALIGNMENT_REGION ;
  rdf:value "KQKLILAYSGGLDTSVAIKWL--SKDYDVV" .

example:AN_ALIGNMENT_REGION
  a faldo:Region ;
  faldo:begin example:AN_ALIGNMENT_REGION_BEGIN ;
  faldo:end example:AN_ALIGNMENT_REGION_END .

example:AN_ALIGNMENT_REGION_BEGIN
  a faldo:ExactPosition ;
  faldo:reference example:P1-seq ;
  faldo:position 1 .

example:AN_ALIGNMENT_REGION_END
  a faldo:ExactPosition ;
  faldo:reference example:P1-seq ;
  faldo:position 28 .
```

Figure 5. Example protein sequence/signature match in RDF syntax.

Protein sequence/signature matches in RDF syntax

HAMAP SPARQL rules require sequence/signature match data in an RDF format.

Figure 4 shows an RDF representation of the sequence/signature match of the example protein ‘P1’ (Figure 4) and the HAMAP signature MF_00005. The core information is a triple that states that the protein (example:P1) matches the signature (signature:MF_00005).

For positional annotations, the rule needs the start and end positions of the match region on the sequence, as well as the alignment between sequence and signature. We describe this information with the EDAM and FALDO ontologies and use the alignment format returned by the pfsearchV3 [20] and InterProScan [10] software.

A HAMAP rule specifies the sequence positions of feature annotations – such as active sites or binding regions – with respect to one or more experimentally characterized ‘template’ sequences in UniProtKB/Swiss-Prot. The rule engine therefore requires the alignment(s) of the template sequence(s) to the rule’s signature as input, and uses these to determine the corresponding positions on the template(s) and target sequence. A HAMAP rule may additionally require that the matching discrete position or range on the target sequence correspond to a specified amino acid or sequence motif, e.g. to check that an active site has the expected amino acid. This functionality can be implemented either in standard SPARQL 1.1 using the REPLACE, STRLEN and CONCAT functions (see Supplementary Figure 1 for an example), or via a custom SPARQL function (an example Java function for RDF stores that extends the Apache Jena ARQ SPARQL engine is given in Supplementary Figure 2). We distribute the template sequence/signature alignments that are required for rule application together with the rules on our FTP site (at ftp://ftp.expasy.org/databases/hamap/hamap_sparql.tar.gz).

Table 1. Simple protein-annotation associations of HAMAP rule MF_00005 for UniProtKB entry B1YJ35.

Protein	Annotation
uniprot:B1YJ35	"GO:0004055"
uniprot:B1YJ35	"GO:0005524"
uniprot:B1YJ35	"GO:0006526"
uniprot:B1YJ35	"GO:0005737"
uniprot:B1YJ35	"ec:6.3.4.5"
uniprot:B1YJ35	"rhea:10932"

Simplifying the output from HAMAP rules for other annotation pipelines

HAMAP rules provide functional annotation in the form of free text and using controlled vocabularies and ontologies developed by UniProt and others. These include the Gene Ontology (GO) [21], the Enzyme Classification of the IUBMB ("EC numbers") [22] represented by the ENZYME database [23], and the Rhea database of biochemical reactions [24] based on the ChEBI ontology [25]. Each HAMAP rule provides all annotation fields required in UniProtKB. For users requiring only a subset of these annotations — such as enzymatic reactions described using Rhea, or protein functions, processes and cellular components described using GO — it is possible to translate only the desired annotation types into SPARQL queries. We can also modify the CONSTRUCT/INSERT block of the queries to return the results as simple protein-annotation associations (see Table 1). This tabular result format can easily be loaded into a relational database or JSON-based document store and requires no further investment in a Semantic Web technology stack.

Results

Validation

We have tested the approach of executing rule-based annotation with a generic SPARQL engine with the data from the HAMAP and UniProtKB/Swiss-Prot releases 2019_10. We translated the HAMAP rules into SPARQL CONSTRUCT queries and the protein sequences into the RDF format described in Figure 4. We generated the RDF representation of the sequence/signature matches, as illustrated in Figure 5, directly from a relational database containing the results of pfsearchV3 scans of UniProtKB/Swiss-Prot versus HAMAP for our internal HAMAP release pipeline. Other groups could achieve the same result by scanning their protein sequences with InterProScan and converting the XML result files into the described RDF format for sequence/signature matches. We provide an XSLT stylesheet for this conversion in https://github.com/sib-swiss/HAMAP-SPARQL/blob/master/src/main/xlst/interproToRdf.xslt. Or use pftoolsV3.2 which has the required RDF format as one of it's output options.

We tested two different open-source SPARQL engines (Virtuoso RDF 7.2 and Apache Jena TDB-2 3.13.1) to execute our rules and validated the generated annotations by comparing them to those obtained from our custom platform. This platform, implemented in Scala/Java, uses as input files protein entries in FASTA format and HAMAP rules in their custom text format to generate annotations in UniProtKB format (text, XML or RDF). The RDF data generated by the different systems was loaded into separate named graphs of an RDF database for comparisons using SPARQL queries to search for annotations unique to any of the three runs (see example query in Figure 6). The existing custom HAMAP annotation pipeline and each of

```
PREFIX rdfs: <http://www.w3.org/2000/01/rdf-schema#>
SELECT
    ?protein
    ?goOrKeyword
WHERE {
  GRAPH <http://example.org/hamap_original/> {
    ?protein up:classifiedWith ?goOrKeyword
  }
  MINUS {
    GRAPH <http://example.org/output-new/> {
      {
        ?protein
          up:classifiedWith
            ?goOrKeyword
      } UNION {
        ?protein
          up:classifiedWith/rdfs:subClassOf
            ?goOrKeyword
      }
    }
  }
}
```

Figure 6. Example query for comparison of annotations generated by the different systems, taking into account if a system inserts a whole go or uniprot keyword hierarchy or only leaf nodes.

the two SPARQL engines generated identical annotations, except for those that depend on external sequence analysis methods and the evaluation of HAMAP rule precedence, which we did not implement here as described in section 2.1.

On a laptop with 8 cores, it takes about 4 minutes to scan a small *E. coli* proteome with the HAMAP signatures using pftoolsV3.2, and 1 minute to execute the HAMAP rules with Apache Jena TDB2 (see the tutorial for instructions). This shows that the sequence/signature scanning step is the bottleneck in our system. Both steps, scanning and rule execution, could be run in an embarrassingly parallel fashion. A further optimization in a HPC setting would be to avoid HTTP communication by running the SPARQL query reader and processor in the same process.

An additional small benefit of the SPARQL representation is that SPARQL queries can be serialized in RDF and loaded into a SPARQL engine. We set-up a server with our rules at https://hamap.expasy.org/sparql. This allows us to perform quality assurance on our rules by running analytical queries across them and SPARQL enpoints of other life science resources.

Discussion

Protein function annotation pipelines based on SPARQL

Here we have developed a SPARQL representation of HAMAP annotation rules that allows other groups with basic knowledge of this widespread standard technology to incorporate HAMAP in their own genome and proteome annotation pipelines. SPARQL can express all features of complex HAMAP rules, including the logic required for positional annotations, while freely available SPARQL engines provide a means to execute HAMAP rules without recourse to specialized software. This work demonstrates the feasibility of adopting SPARQL as a means to integrate existing functional annotation pipelines for genome sequencing projects. This applies not only to expert curated rules from HAMAP and other systems, but also anno-

A)

```
base <http://example.org/rnacentral/>
prefix SO: <http://purl.obolibrary.org/obo/SO_>
prefix rfam: <http://rfam.xfam.org/family/>
<URS0000638944>
  a SO:0000356 ;
  rdf:value """CUAGACCGAAGCUGCCAAGGUGCGUGAUCC
CUCGGUGAUGCCUUGAGUGUUGCUUCGCCAAAAAACAACCACACG
GCCUAGCCGAAUUUCUCAUU""" ;
  rdfs:seeAlso rfam:RF00003 .
```

B)

```
base <http://example.org/rnacentral/>
prefix SO: <http://purl.obolibrary.org/obo/SO_>
prefix GO: <http://purl.obolibrary.org/obo/GO_>
prefix rfam:<http://rfam.xfam.org/family/>
CONSTRUCT {
  ?rna SO:associated_with GO:0005685
}
WHERE {
  ?rna a SO:0000356 ;
  rdfs:seeAlso rfam:RF00003 .
}
```

Figure 7. (A) Hypothetical triples to describe a sequence entry from RNAcentral.org that is a member of the Rfam RNA family RF00003 (U1 spliceosomal RNA family). (B) Hypothetical rule associating RF00003 to the GO term GO:0005685 (definition: "A ribonucleoprotein complex that contains small nuclear RNA U1.").

tation rules generated by automated approaches such as deep learning [26, 27], which require a feature vector to be expressed as an RDF triple as shown by LOD4ML (http://lod4ml.org). SPARQL can also be adopted by those without access to specialized RDF triple stores by using a SPARQL to SQL mapping (such as that provided by any of the R2RML tools, see https://www.w3.org/TR/r2rml/) to execute SPARQL rules directly against data stored in a relational database. The main weakness of SPARQL is that, like many generic query engines, it tends to be computationally more expensive than a custom solution, but we have seen significant progress in the optimization of SPARQL engines over the past years [28].

An approach that is extensible to any domain of biology

While we have limited our demonstration to the use of SPARQL queries to formalize and execute protein annotation rules from HAMAP, there is nothing that ties the SPARQL approach to a particular domain of biology. Complete genome annotation requires identification and functional annotation of RNAs as well as proteins, and Figure 7 provides a demonstration of how that annotation could be provided by SPARQL. Here a hypothetical SPARQL rule specifies functional (GO) annotation for an RNA sequence of RNAcentral [29] that is a member of the U1 spliceosomal RNA family as defined by Rfam [30].

The development of annotation rules for a given domain across different groups will require community standards for the representation of the relevant domain-specific annotation types. In this work we have used the RDF vocabularies of UniProt, which allowed us to easily compare the results of the SPARQL approach to those of our existing HAMAP rule annotation pipeline. As other appropriate community ontologies become available, our queries and SPARQL rules can be easily adapted.

Further work

We plan to further extend our implementation of HAMAP rules using SPARQL to include external method calls and deal with rule precedence (see Section 2.1), and also develop a SPARQL representation for PROSITE, which provides protein domain annotation via a custom pipeline, ScanProsite (at https://prosite.expasy.org/scanprosite/) [31]. HAMAP and PROSITE are two of the main components of the UniRule system of UniProt, which provides automatic annotation for unreviewed entries of UniProtKB/TrEMBL [32], and the approach described here could be extended to the entire UniRule system. The UniProt data model was recently extended to allow enzyme annotation using biochemical reaction data from the Rhea database [33], which will further extend the scope of HAMAP SPARQL rules to more specialized applications – such as the creation and annotation of draft networks of metabolic reactions [34, 35].

Conclusion

Acknowledgements

We thank Dr Marco Pagni of the SIB Swiss Institute of Bioinformatics for interesting discussions and critical reading of the manuscript.

Funding

HAMAP activities at the SIB are supported by the Swiss Federal Government through the State Secretariat for Education, Research and Innovation SERI, and the Swiss National Science Foundation (SNSF). The development of the HAMAP SPARQL rules was also supported by the ELIXIR Implementation study on "A microbial metabolism resource for Systems Biology". Funding for open access charge: SERI.

Conflict of Interest: none declared.

References

1. Lewin, H.A., et al. Earth BioGenome Project: Sequencing life for the future of life. *Proc Natl Acad Sci U S A* 2018;115(17):4325-4333.
2. Mukherjee, S., et al. 1,003 reference genomes of bacterial and archaeal isolates expand coverage of the tree of life. *Nat Biotechnol* 2017;35(7):676-683.
3. Paez-Espino, D., et al. Uncovering Earth's virome. Nature 2016;536(7617):425-430.
4. Thompson, L.R., et al. A communal catalogue reveals Earth's multiscale microbial diversity. *Nature* 2017;551(7681):457-463.
5. Tighe, S., et al. Genomic Methods and Microbiological Technologies for Profiling Novel and Extreme Environments for the Extreme Microbiome Project (XMP). *J Biomol Tech* 2017;28(1):31-39.
6. Meyer, F., et al. MG-RAST version 4-lessons learned from a decade of low-budget ultra-high-throughput metagenome analysis. *Brief Bioinform* 2017.
7. Overbeek, R., et al. The SEED and the Rapid Annotation of microbial genomes using Subsystems Technology (RAST). *Nucleic Acids Res* 2014;42(Database issue):D206-214.
8. Chen, I.A., et al. IMG/M: integrated genome and metagenome comparative data analysis system. *Nucleic Acids Res* 2017;45(D1):D507-D516.
9. Haft, D.H., et al. RefSeq: an update on prokaryotic genome

annotation and curation. *Nucleic Acids Res*, Volume 46, Issue D1, 4 January 2018, Pages D851–D860,
10. Mitchell, A.L., et al. InterPro in 2019: improving coverage, classification and access to protein sequence annotations. *Nucleic Acids Res* 2019;47(D1):D351-D360.
11. Haft, D.H., et al. TIGRFAMs and Genome Properties in 2013. *Nucleic Acids Res* 2013;41(Database issue):D387-395.
12. Pedruzzi, I., et al. HAMAP in 2015: updates to the protein family classification and annotation system. *Nucleic Acids Res* 2015;43(Database issue):D1064-D1070.
13. Bolleman, J.T., et al. FALDO: a semantic standard for describing the location of nucleotide and protein feature annotation. *J Biomed Semantics* 2016;7:39.
14. Zerbino, D.R., et al. *Ensembl* 2018. Nucleic Acids Res 2018;46(D1):D754-D761.
15. Kersey, P.J., et al. Ensembl Genomes 2018: an integrated omics infrastructure for non-vertebrate species. *Nucleic Acids Res* 2018;46(D1):D802-D808.
16. Ison, J., et al. EDAM: an ontology of bioinformatics operations, types of data and identifiers, topics and formats. *Bioinformatics* 2013;29(10):1325-1332.
17. Chibucos, M.C., et al. Standardized description of scientific evidence using the Evidence Ontology (ECO). *Database (Oxford)* 2014;2014.
18. Petersen, T.N., et al. SignalP 4.0: discriminating signal peptides from transmembrane regions. *Nat Methods* 2011;8(10):785-786.
19. Sonnhammer, E.L., von Heijne, G. and Krogh, A. A hidden Markov model for predicting transmembrane helices in protein sequences. *Proc Int Conf Intell Syst Mol Biol* 1998;6:175-182.
20. Schuepbach, T., et al. pfsearchV3: a code acceleration and heuristic to search PROSITE profiles. *Bioinformatics* 2013;29(9):1215-1217.
21. The Gene Ontology Consortium. The Gene Ontology Resource: 20 years and still GOing strong. *Nucleic Acids Res* 2019;47(D1):D330-D338.
22. McDonald, A.G., Boyce, S. and Tipton, K.F. ExplorEnz: the primary source of the IUBMB enzyme list. *Nucleic Acids Res* 2009;37(Database issue):D593-597.
23. Bairoch, A. The ENZYME database in 2000. *Nucleic Acids Res* 2000;28(1):304-305.
24. Lombardot, T., et al. Updates in Rhea: SPARQLing biochemical reaction data. *Nucleic Acids Res* 2018;47(D1):D596-D600.
25. Hastings, J., et al. ChEBI in 2016: Improved services and an expanding collection of metabolites. *Nucleic Acids Res* 2016;44(D1):D1214-1219.
26. Fa, R., et al. Predicting human protein function with multi-task deep neural networks. *PLoS One* 2018;13(6):e0198216.
27. Kulmanov, M., et al. DeepGO: predicting protein functions from sequence and interactions using a deep ontology-aware classifier. *Bioinformatics* 2018;34(4):660-668.
28. Schmidt, M., Meier, M. and Lausen, G. Foundations of SPARQL query optimization. *Proceedings of the 13th International Conference on Database Theory* 2010:4-33.
29. The RNAcentral Consortium. RNAcentral: a comprehensive database of non-coding RNA sequences. *Nucleic Acids Res* 2017;45(D1):D128-D134.
30. Kalvari, I., et al. Rfam 13.0: shifting to a genome-centric resource for non-coding RNA families. *Nucleic Acids Res* 2018;46(D1):D335-D342.
31. Sigrist, C.J., et al. New and continuing developments at PROSITE. *Nucleic Acids Res* 2013;41(Database issue):D344-347.
32. The UniProt Consortium. UniProt: a worldwide hub of protein knowledge. *Nucleic Acids Res* 2019;47(D1):D506-D515.
33. Morgat, A., et al. Enzyme annotation in UniProtKB using Rhea. *Bioinformatics* 2019;btz817.
34. Faria, J.P., et al. Methods for automated genome-scale metabolic model reconstruction. *Biochem Soc Trans* 2018;46(4):931-936.
35. Moretti, S., et al. MetaNetX/MNXref–reconciliation of metabolites and biochemical reactions to bring together genome-scale metabolic networks. *Nucleic Acids Res* 2016;44(D1):D523-526.

Click here to access/download
Supplementary Material
bolleman_supplement.tex

Click here to access/download
Supplementary Material
bolleman_supplement.pdf
