# Supplementary material for: HAMAP as SPARQL rules—A portable annotation pipeline for genomes and proteomes
Source: Gigascience. 2020 Feb 8;9(2):giaa003. doi: 10.1093/gigascience/giaa003 (PMC7007698; doi:10.1093/gigascience/giaa003)
Supplement: giaa003_GIGA-D-19-00242_Revision_2 [file giaa003_giga-d-19-00242_revision_2.pdf]

GigaScience

HAMAP as SPARQL rules - A portable annotation pipeline for genomes and proteomes

--Manuscript Draft--

**Manuscript Number:**	GIGA-D-19-00242R2
**Full Title:**	HAMAP as SPARQL rules - A portable annotation pipeline for genomes and proteomes
**Article Type:**	Research
**Funding Information:**	Staatssekretariat für Bildung, Forschung und Innovation (CH) — Dr Alan Bridge
**Abstract:**	Genome and proteome annotation pipelines are generally custom built and therefore not easily reusable by other groups, which leads to duplication of effort, increased costs, and suboptimal results. One cost-effective way to increase the data quality in public databases is to encourage the adoption of annotation standards and technological solutions that enable the sharing of biological knowledge and tools for genome and proteome annotation. We have translated the rules of our HAMAP proteome annotation pipeline to queries in the W3C standard SPARQL 1.1 syntax and applied them with two off-the-shelf SPARQL engines to UniProtKB/Swiss-Prot protein sequences described in RDF format. This approach is applicable to any genome or proteome annotation pipeline and greatly simplifies their reuse. HAMAP SPARQL rules and documentation are freely available for download from the HAMAP FTP site ftp://ftp.expasy.org/databases/hamap/hamap_sparql.tar.gz under a CC-BY-ND 4.0 license. The annotations generated by the rules are under the CC-BY 4.0 license.
**Corresponding Author:**	Jerven Tjalling Bolleman<br>Swiss Institute of Bioinformatics Geneva<br>Geneva, GE SWITZERLAND
**Corresponding Author Secondary Information:**	
**Corresponding Author's Institution:**	Swiss Institute of Bioinformatics Geneva
**Corresponding Author's Secondary Institution:**	
**First Author:**	Jerven Tjalling Bolleman
**First Author Secondary Information:**	
**Order of Authors:**	Jerven Tjalling Bolleman
	Eduoard de Castro
	Delphine Baratin
	Sebastien Gehant
	Beatrice A Cuche
	Andrea H Auchincloss
	Elisabeth Coudert
	Chantal Hulo
	Patrick Masson
	Ivo Pedruzzi
	Catherine Rivoire
	Ioannis Xenarios
	Nicole Redaschi
	Alan Bridge
**Order of Authors Secondary Information:**	

Powered by Editorial Manager® and ProduXion Manager® from Aries Systems Corporation

**Response to Reviewers:**	Dear Editors,<br><br>We have included the DOI as requested. I also made a single change to the first reference which was pointing to a compressed file on the HAMAP ftp site. This was changed to a directory as the contents of the compressed file are now available there directly.<br><br>Regards,<br>Jerven
**Additional Information:**	
**Question**	**Response**
Are you submitting this manuscript to a special series or article collection?	No
**Experimental design and statistics**<br><br>Full details of the experimental design and statistical methods used should be given in the Methods section, as detailed in our Minimum Standards Reporting Checklist. Information essential to interpreting the data presented should be made available in the figure legends.<br><br>Have you included all the information requested in your manuscript?	No
If not, please give reasons for any omissions below.<br><br>as follow-up to "**Experimental design and statistics**<br><br>Full details of the experimental design and statistical methods used should be given in the Methods section, as detailed in our Minimum Standards Reporting Checklist. Information essential to interpreting the data presented should be made available in the figure legends.<br><br>Have you included all the information requested in your manuscript?<br><br>"	Not applicable
**Resources**	Yes

A description of all resources used, including antibodies, cell lines, animals and software tools, with enough information to allow them to be uniquely identified, should be included in the Methods section. Authors are strongly encouraged to cite Research Resource Identifiers (RRIDs) for antibodies, model organisms and tools, where possible.<br><br>Have you included the information requested as detailed in our Minimum Standards Reporting Checklist?	
**Availability of data and materials**<br><br>All datasets and code on which the conclusions of the paper rely must be either included in your submission or deposited in publicly available repositories (where available and ethically appropriate), referencing such data using a unique identifier in the references and in the "Availability of Data and Materials" section of your manuscript.<br><br>Have you have met the above requirement as detailed in our Minimum Standards Reporting Checklist?	Yes

Powered by Editorial Manager® and ProduXion Manager® from Aries Systems Corporation

Manuscript

Click here to access/download;Manuscript;bolleman.tex

Placeholder for OUP logo oup.pdf

Placeholder for journal logo gigascience-logo.pdf

GigaScience, 2017, 1–7

doi: xx.xxxx/xxxx
Manuscript in Preparation
Paper

PAPER

HAMAP as SPARQL rules – A portable annotation pipeline for genomes and proteomes

Jerven Bolleman[1,*], Edouard de Castro[1], Delphine Baratin[1], Sebastien Gehant[1], Beatrice A. Cuche[1], Andrea H. Auchincloss[1], Elisabeth Coudert[1], Chantal Hulo[1], Patrick Masson[1], Ivo Pedruzzi[1], Catherine Rivoire[1], Ioannis Xenarios[1,2], Nicole Redaschi[1] and and Alan Bridge[1]

[1]Swiss-Prot Group, SIB Swiss Institute of Bioinformatics, CMU, 1 rue Michel-Servet, CH-1211 Geneva 4, Switzerland and [2]CHUV/LICR, Agora Centre, CH-1005 Lausanne, Switzerland

[*]jerven.bolleman@sib.swiss, hamap@sib.swiss

Abstract

Genome and proteome annotation pipelines are generally custom built and not easily reusable by other groups. This leads to duplication of effort, increased costs, and suboptimal annotation quality. One way to address these issues is to encourage the adoption of annotation standards and technological solutions that enable the sharing of biological knowledge and tools for genome and proteome annotation.
Here we demonstrate one approach to generate portable genome and proteome annotation pipelines that users can run without recourse to custom software. This proof of concept uses our own rule-based annotation pipeline HAMAP, which provides functional annotation for protein sequences to the same depth and quality as UniProtKB/Swiss-Prot, and the World Wide Web Consortium (W3C) standards Resource Description Framework (RDF) and SPARQL (a recursive acronym for the SPARQL Protocol and RDF Query Language). We translate complex HAMAP rules into the W3C standard SPARQL 1.1 syntax, and then apply them to protein sequences in RDF format using freely available SPARQL engines. This approach supports the generation of annotation that is identical to that generated by our own in-house pipeline, using standard, off the shelf solutions, and is applicable to any genome or proteome annotation pipeline.
HAMAP SPARQL rules are freely available for download from the HAMAP FTP site [1] under the CC-BY-ND 4.0 license. The annotations generated by the rules are under the CC-BY 4.0 license. A tutorial and supplementary code to use HAMAP as SPARQL are available on GitHub [2] and general documentation about HAMAP can be found on the HAMAP website [3].

Key words: Protein, Function, Prediction, SPARQL

Introduction

Continuing technological advances have reduced the costs of DNA sequencing enormously in recent years, leading to an explosion in the number of available whole genome and metagenome sequences from all branches of the tree of life [4, 5, 6, 7, 8]. This wealth of sequence data presents exciting opportunities for experimental and computational research into the evolution and functional capacities of individual organisms and the communities they form, but fully exploiting this data will require complete and accurate functional annotation of these genome and metagenome sequences. Resources for genome annotation such as RAST/MG-RAST [9, 10], IMG/M [11], the NCBI genome annotation pipeline [12], InterPro [13], TIGRFAMS [14], and HAMAP [15] exploit information from experimentally characterized sequences to infer functions for uncharacterized homologs. While the underlying principles of these resources are undoubtedly very similar, a lack of shared annotation standards and a suitable shared technical framework for annotation hamper efforts to use and combine them.

In this work, we use the HAMAP system to demonstrate technical solutions that could facilitate the combination and reuse of functional genome annotation systems from any provider. HAMAP classifies and annotates protein sequences using a collection of expert-curated protein family signatures and annotation rules. Swiss-Prot curators build HAMAP rules as part of an integrated workflow that includes curation of experimentally characterized template entries in UniProtKB/Swiss-Prot, as well as curation of the associated rule and protein family signature (encoded as a generalized profile). HAMAP rules annotate family members to the same level of detail and quality as the expert curated UniProtKB/Swiss-Prot records on which they are based, combining family mem-

```
prefix rdf:
 <http://www.w3.org/1999/02/22-rdf-syntax-ns#>
prefix rdfs:
 <http://www.w3.org/2000/01/rdf-schema#>
prefix up:
 <http://purl.uniprot.org/core/>
prefix taxon:
 <http://purl.uniprot.org/taxonomy/>
prefix ec:
 <http://purl.uniprot.org/enzyme/>
prefix GO:
 <http://purl.obolibrary.org/obo/GO_>
prefix ECO:
 <http://purl.obolibrary.org/obo/ECO_>
prefix faldo:
 <http://biohackathon.org/resource/faldo#>
prefix rule:
 <http://purl.uniprot.org/hamap-rule/>
prefix signature:
 <http://purl.uniprot.org/hamap/>
prefix edam:
 <http://edamontology.org/>
prefix example:
 <URL space of your protein sequences>
```

Figure 1. RDF namespace declarations for prefixes used in other Figures.

bership and residue dependencies to ensure a high degree of specificity [15].

The current implementation of HAMAP uses a custom rule format and annotation engine that are not easy to integrate into external pipelines. The HAMAP-Scan web service [16] is a good alternative for small research projects, but large genome sequencing projects cannot depend on external web services to process large amounts of data. Our goal here was to develop a generic HAMAP rule format and annotation engine that is easily portable by external HAMAP users, using standard technologies that developers of other genome annotation pipelines could also adopt. To achieve this we have developed a representation of HAMAP annotation rules using the W3C standard SPARQL 1.1 syntax. SPARQL [17] is a query language for RDF [18], a core Semantic Web technology from the W3C. Our implementation allows users to apply HAMAP rules in SPARQL syntax to annotate protein sequences expressed as RDF using off-the-shelf SPARQL engines - without any need for a custom pipeline. If other annotation system providers were to adopt the same approach, it would then be possible to share and combine the annotation rules from multiple systems, execute them with any SPARQL engine, and compare the results.

Methods

To use a generic SPARQL engine to execute rule-based protein sequence annotation, we need the following input data: a) annotation rules in SPARQL syntax, b) protein sequence records in RDF syntax, and c) protein sequence/signature matches in RDF syntax, including alignment information for positional annotations.

To keep the examples given in the Figures short, we provide all RDF namespace prefixes declarations in Figure 1 and omit these from subsequent Figures. We use the UniProt core ontology and other ontologies used by UniProt, such as the Feature Annotation Location Description Ontology (FALDO) [19], which is also used in the RDF of Ensembl [20] and Ensembl Genomes [21], to describe sequence positions, and the EDAM ontology [22] to describe sequence/signature matches.

```
CONSTRUCT {
  ?target up:classifiedWith GO:0004055 , GO:0005524 .
}
WHERE {
  ?target a up:Protein ;
          rdfs:seeAlso signature:MF_00005 ;
          up:sequence ?targetSequence ;
          up:organism ?organism .

  VALUES ?bacteriaORarchaea {taxon:2 taxon:2157}
  ?organism rdfs:subClassOf+ ?bacteriaORarchaea  .

  MINUS { ?targetSequence up:fragment [] }
}
```

Figure 2. Part of the HAMAP rule for signature MF_00005 as a SPARQL CONSTRUCT query.

```
CONSTRUCT {
  # annotation statements
  ?target up:classifiedWith GO:0004055 , GO:0005524 .
  # metadata statements
  _:1 a              rdf:Statement ;
      rdf:subject    ?target ;
      rdf:predicate  up:classifiedWith ;
      rdf:object     GO:0004055 ;
      up:attribution _:3 .

  _:2 a              rdf:Statement ;
      rdf:subject    ?target ;
      rdf:predicate  up:classifiedWith ;
      rdf:object     GO:0005524 ;
      up:attribution _:3 .

  _:3 up:source       rule:MF_00005 ;
      up:evidence     ECO:256 .
}
```

Figure 3. SPARQL CONSTRUCT block of Fig. 2 extended with metadata expressed as RDF reification quads.

HAMAP annotation rules in SPARQL syntax

A HAMAP annotation rule consists of two parts: 1) the annotations, and 2) a set of conditions that must be satisfied in order to apply those annotations. The rule annotations can be expressed either by a CONSTRUCT block that returns the annotations as RDF triples or by an INSERT block that inserts these triples directly into an RDF store, while the rule conditions can be expressed by the WHERE clause of a SPARQL query. Figure 2 shows part of the HAMAP rule for the signature MF_00005 as a SPARQL query. The CONSTRUCT block generates two annotations consisting of RDF triples for two Gene Ontology (GO) terms, providing that all conditions defined in the WHERE clause are satisfied. The conditions here are that the target must be a complete protein sequence, of bacterial or archaeal origin, and a member of the HAMAP family MF_00005 (i.e. matching the corresponding family signature).

Figure 3 shows how the CONSTRUCT block of Figure 2 can be extended to generate metadata for provenance and evidence for each annotation that the rule generates. We attribute the annotations to the HAMAP rule (MF_00005) and describe the type of the evidence with a value from the Evidence Code Ontology (ECO) [23]. We link the attribution to the annotations via RDF reification quads, which is verbose but is understood by all RDF syntaxes and data stores.

```
example:P1
  a up:Protein ;
  up:sequence example:P1-seq ;
  up:organism taxon:83333 .

example:P1-seq
  a up:Simple_Sequence ;
  rdf:value """MTKQKLILAYSGGLDTSVAIKWLSKDYDVVAL
CMDVGEGKDLSVIKEKALLVGAIESIVLDVKDEFANDFVLPALQYGA
HYEGAYPLISALSRPLIAEKLVEVAHAQGATAVAHGCTGKGNDQVRF
EVSVAALDPSLEVIAPVREWKWSREEEIAYAKENNVPIPINLNSPYS
IDMNLWGRSNECGVLENPWTEPPQDAYALTVAPEDAPDQAEEVIIGF
EAGVPVSINGTAYPLAKLITELNIIAGAHGVGRIDHVENRLVGIKSR
EVYECPGATVLLKAHAALETITLTKDVAHFKPILSKQYAETIYNGLF
HAPLTKGLKAFLTATQQDVTGEVRVKLYKGNATVTGRQSAVSLYDEK
LATYTKEDAFDHEAAKGFIKLHGLAISTHASVHRQEGVKK""" .
```

Figure 4. Example protein record in an RDF format suitable for HAMAP SPARQL rules.

The original HAMAP rule implementation has two features that we have not yet implemented in this work. The first is the ability to call sequence analysis methods such as SignalP [24] and TMHMM [25] for the annotation of signal and transmembrane regions, which is not implemented here as these methods may not be available to external users. The second is precedence relationships between HAMAP rules, which are complex and apply to relatively few rules.

Protein sequence records in RDF syntax

HAMAP SPARQL rules require protein sequence records in a simple RDF format. Figure 4 shows an example protein record with the identifier 'P1' (example:P1). The rules require an identifier for the sequence (example:P1-seq) and the organism as an NCBI taxonomy identifier (taxon:83333). The actual protein sequence is provided as an IUPAC amino acid encoded string (in the rdf:value predicate of example:P1-seq) for positional annotations.

Protein sequence/signature matches in RDF syntax

HAMAP SPARQL rules require sequence/signature match data in an RDF format.

Figure 4 shows an RDF representation of the sequence/signature match of the example protein 'P1' (Figure 4) and the HAMAP signature MF_00005. The core information is a triple that states that the protein (example:P1) matches the signature (signature:MF_00005).

For positional annotations, the rule needs the start and end positions of the match region on the sequence, as well as the alignment between sequence and signature. We describe this information with the EDAM and FALDO ontologies and use the alignment format returned by the PfTools v3 [26] and InterProScan [13] software.

A HAMAP rule specifies the sequence positions of feature annotations – such as active sites or binding regions – with respect to one or more experimentally characterized 'template' sequences in UniProtKB/Swiss-Prot. The rule engine therefore requires the alignment(s) of the template sequence(s) to the rule's signature as input, and uses these to determine the corresponding positions on the template(s) and target sequence. A HAMAP rule may additionally require that the matching discrete position or range on the target sequence correspond to a specified amino acid or sequence motif, e.g. to check that an active site has the expected amino acid. This functionality can be

```
example:P1
  rdfs:seeAlso signature:MF_00005 ;
  up:sequence example:P1-seq .

example:AN_ALIGNMENT_OPERATION
  a edam:operation_0300 ;
  edam:has_input signature:MF_00005 .

example:AN_ALIGNMENT
  a edam:data_0869 ;
  edam:is_output_of example:AN_ALIGNMENT_OPERATION ;
  faldo:location example:AN_ALIGNMENT_REGION ;
  rdf:value "KQKLILAYSGGLDTSVAIKWL--SKDYDVV" .

example:AN_ALIGNMENT_REGION
  a faldo:Region ;
  faldo:begin example:AN_ALIGNMENT_REGION_BEGIN ;
  faldo:end example:AN_ALIGNMENT_REGION_END .

example:AN_ALIGNMENT_REGION_BEGIN
  a faldo:ExactPosition ;
  faldo:reference example:P1-seq ;
  faldo:position 1 .

example:AN_ALIGNMENT_REGION_END
  a faldo:ExactPosition ;
  faldo:reference example:P1-seq ;
  faldo:position 28 .
```

Figure 5. Example protein sequence/signature match in RDF syntax.

implemented either in standard SPARQL 1.1 using the REPLACE, STRLEN and CONCAT functions (see Supplementary Figure 1 for an example), or via a custom SPARQL function (an example Java function for RDF stores that extends the Apache Jena ARQ SPARQL engine is given in Supplementary Figure 2). We distribute the template sequence/signature alignments that are required for rule application together with the rules on our FTP site [1].

Simplifying the output from HAMAP rules for other annotation pipelines

HAMAP rules provide functional annotation in the form of free text and using controlled vocabularies and ontologies developed by UniProt and others. These include the Gene Ontology (GO) [27], the Enzyme Classification of the IUBMB ("EC numbers") [28] represented by the ENZYME database [29], and the Rhea database of biochemical reactions [30] based on the ChEBI ontology [31]. Each HAMAP rule provides all annotation fields required in UniProtKB. For users requiring only a subset of these annotations — such as enzymatic reactions described using Rhea, or protein functions, processes and cellular components described using GO — it is possible to translate only the desired annotation types into SPARQL queries. We can also modify the CONSTRUCT/INSERT block of the queries to return the results as simple protein-annotation associations (see Table 1). This tabular result format can easily be loaded into a relational database or JSON-based document store and requires no further investment in a Semantic Web technology stack.

Results

Table 1. Simple protein-annotation associations of HAMAP rule MF_00005 for UniProtKB entry B1YJ35.

Protein	Annotation
uniprot:B1YJ35	"GO:0004055"
uniprot:B1YJ35	"GO:0005524"
uniprot:B1YJ35	"GO:0006526"
uniprot:B1YJ35	"GO:0005737"
uniprot:B1YJ35	"ec:6.3.4.5"
uniprot:B1YJ35	"rhea:10932"

Validation

We have tested the approach of executing rule-based annotation with a generic SPARQL engine with the data from the HAMAP and UniProtKB/Swiss-Prot releases 2019_10. We translated the HAMAP rules into SPARQL CONSTRUCT queries and the protein sequences into the RDF format described in Figure 4. We generated the RDF representation of the sequence/signature matches, as illustrated in Figure 5, directly from a relational database containing the results of PfTools v3.1 scans of UniProtKB/Swiss-Prot versus HAMAP for our internal HAMAP release pipeline. Other groups could achieve the same result by scanning their protein sequences with PfTools v3.2 [32], which has a new output option for RDF format, or with InterProScan and converting the XML result files into RDF format with the XSLT stylesheet that we provide for this purpose [33].

We tested two different open-source SPARQL engines (Virtuoso RDF 7.2 and Apache Jena TDB2 3.13.1) to execute our rules and validated the generated annotations by comparing them to those obtained from our custom platform. This platform, implemented in Scala/Java, uses as input files protein entries in FASTA format and HAMAP rules in their custom text format to generate annotations in UniProtKB format (text, XML or RDF). The RDF data generated by the different systems was loaded into separate named graphs of an RDF database for comparisons using SPARQL queries to search for annotations unique to any of the three runs (see example query in Figure 6). The existing custom HAMAP annotation pipeline and each of the two SPARQL engines generated identical annotations, except for those that depend on external sequence analysis methods and the evaluation of HAMAP rule precedence, which we did not implement here as described in section 2.1.

On a laptop with 8 cores, it takes about 4 minutes to scan a small *E. coli* proteome with the HAMAP signatures using PfTools v3.2 or InterProScan software, and 1 minute to execute the HAMAP rules with Apache Jena TDB2 (see the instructions in the tutorial [2]). This shows that the sequence/signature scanning step is the bottleneck in our system. Both steps, scanning and rule execution, could be run in an embarrassingly parallel fashion. A further optimization for high-performance computing would be to avoid HTTP communication by running the SPARQL query reader and processor in the same process.

An additional small benefit of the SPARQL representation is that SPARQL queries can be serialized in RDF and loaded into a SPARQL engine. We set-up a server with our rules [34] that allows us to perform quality assurance on our rules by running analytical queries across them and SPARQL enpoints of other life science resources.

Discussion

```
PREFIX rdfs: <http://www.w3.org/2000/01/rdf-schema#>
SELECT ?protein ?goOrKeyword
WHERE {
  GRAPH <http://example.org/hamap_original/> {
    ?protein up:classifiedWith
      ?goOrKeyword
  }
  MINUS {
    GRAPH <http://example.org/output-new/> {
      {
        ?protein up:classifiedWith
          ?goOrKeyword
      } UNION {
        ?protein up:classifiedWith/rdfs:subClassOf
          ?goOrKeyword
      }
    }
  }
}
```

Figure 6. Example query for comparison of annotations generated by the different systems, taking into account whether a system inserts the full GO or UniProt keyword hierarchy or only leaf nodes.

Protein function annotation pipelines based on SPARQL

Here we have developed a SPARQL representation of HAMAP annotation rules that allows other groups with basic knowledge of this widespread standard technology to incorporate HAMAP in their own genome and proteome annotation pipelines. SPARQL can express all features of complex HAMAP rules, including the logic required for positional annotations, while freely available SPARQL engines provide a means to execute HAMAP rules without recourse to specialized software. This work demonstrates the feasibility of adopting SPARQL as a means to integrate existing functional annotation pipelines for genome sequencing projects. This applies not only to expert curated rules from HAMAP and other systems, but also annotation rules generated by automated approaches such as deep learning [35, 36], which require a feature vector to be expressed as an RDF triple as shown by Linked Open Data for Machine Learning (LOD4ML) [37]. SPARQL can also be adopted by those without access to specialized RDF triple stores by using a SPARQL to SQL mapping (such as that provided by any of the R2RML tools [38]) to execute SPARQL rules directly against data stored in a relational database. The main weakness of SPARQL is that, like many generic query engines, it tends to be computationally more expensive than a custom solution, but we have seen significant progress in the optimization of SPARQL engines over the past years [39].

An approach that is extensible to any domain of biology

While we have limited our demonstration to the use of SPARQL queries to formalize and execute protein annotation rules from HAMAP, there is nothing that ties the SPARQL approach to a particular domain of biology. Complete genome annotation requires identification and functional annotation of RNAs as well as proteins, and Figure 7 provides a demonstration of how that annotation could be provided by SPARQL. Here a hypothetical SPARQL rule specifies functional (GO) annotation for an RNA sequence of RNAcentral [40] that is a member of the U1 spliceosomal RNA family as defined by Rfam [41].

The development of annotation rules for a given domain

A)

```
base <http://example.org/rnacentral/>
prefix SO: <http://purl.obolibrary.org/obo/SO_>
prefix rfam: <http://rfam.xfam.org/family/>
<URS0000638944>
  a SO:0000356 ;
  rdf:value """CUAGACCGAAGCUGCCAAGGUGCGUGAUCC
CUCGGUGAUGCCUUGAGUGUUGCUUCGCCAAAAAACAACCACACG
GCCUAGCCGAAUUUCUCAUU""" ;
  rdfs:seeAlso rfam:RF00003 .
```

B)

```
base <http://example.org/rnacentral/>
prefix SO: <http://purl.obolibrary.org/obo/SO_>
prefix GO: <http://purl.obolibrary.org/obo/GO_>
prefix rfam:<http://rfam.xfam.org/family/>
CONSTRUCT {
  ?rna SO:associated_with GO:0005685
}
WHERE {
  ?rna a SO:0000356 ;
  rdfs:seeAlso rfam:RF00003 .
}
```

Figure 7. (A) Hypothetical triples to describe a sequence entry from RNAcentral.org that is a member of the Rfam RNA family RF00003 (U1 spliceosomal RNA family). (B) Hypothetical rule associating RF00003 to the GO term GO:0005685 (definition: "A ribonucleoprotein complex that contains small nuclear RNA U1.").

across different groups will require community standards for the representation of the relevant domain-specific annotation types. In this work we have used the RDF vocabularies of UniProt, which allowed us to easily compare the results of the SPARQL approach to those of our existing HAMAP rule annotation pipeline. As other appropriate community ontologies become available, our queries and SPARQL rules can be easily adapted.

Further work

We plan to further extend our implementation of HAMAP rules using SPARQL to include external method calls and deal with rule precedence (see Section 2.1), and also develop a SPARQL representation for PROSITE, which provides protein domain annotation via a custom pipeline, ScanProsite [42]. HAMAP and PROSITE are two of the main components of the UniRule system of UniProt, which provides automatic annotation for unreviewed entries of UniProtKB/TrEMBL [43], and the approach described here could be extended to the entire UniRule system. The UniProt data model was recently extended to allow enzyme annotation using biochemical reaction data from the Rhea database [44], which will further extend the scope of HAMAP SPARQL rules to more specialized applications – such as the creation and annotation of draft networks of metabolic reactions [45, 46].

Availability of supporting source code and requirements

- Project name: HAMAP
- Project home page: https://hamap.expasy.org
- Other requirements: SPARQL 1.1. compliant RDF store, sequence/signature scanning software (e.g. PfTools v3.2, biotools:pfsearch or InterProScan, RRID:SCR_005829)
- License: CC-BY-ND 4.0
- RRID: SCR_007701

Availability of supporting data

The data sets supporting the results of this article are available in the GigaDB repository[47].

Declarations

List of abbreviations

- ECO: Evidence Code Ontology
- FALDO: Feature Annotation Location Description Ontology
- GO: Gene Ontology
- HAMAP: High-quality Automated and Manual Annotation of Proteins
- LOD4ML: Linked Open Data for Machine Learning
- RDF: Resource Description Framework
- SPARQL: SPARQL Protocol and RDF Query Language
- W3C: World Wide Web Consortium

Ethics approval and consent to participate

Not applicable.

Consent for publication

Not applicable.

Competing interests

The authors declare that they have no competing interests.

Funding

HAMAP activities at the SIB are supported by the Swiss Federal Government through the State Secretariat for Education, Research and Innovation SERI, and the Swiss National Science Foundation (SNSF). The development of the HAMAP SPARQL rules was also supported by the ELIXIR Implementation study on "A microbial metabolism resource for Systems Biology". Funding for open access charge: SERI.

Authors contributions

JTB designed the HAMAP-SPARQL system, implemented the software to convert HAMAP rules to SPARQL, ran the analysis and co-wrote the manuscript. EdC implemented the original HAMAP pipeline and assisted with the translation of the rules to SPARQL. DB implemented software to extract sequence/signature matches in RDF format from an Oracle database. SG helped to optimize SPARQL queries and define the rule data model. BC helped to define the RDF format for sequence/signature matches. AA, EC, CH, PM, IP, CR curate Swiss-Prot entries and HAMAP rules used in this manuscript. IX participated in the planning of the project. NR tested and revised the tutorial and co-wrote the manuscript. AB co-wrote and edited the manuscript. All authors provided critical feedback on the project.

Acknowledgements

We thank Dr Marco Pagni of the SIB Swiss Institute of Bioinformatics for interesting discussions and critical reading of the manuscript.

References

1. HAMAP rules in SPARQL syntax. 2019. ftp://ftp.expasy.org/databases/hamap/sparql/. Accessed 5 Dec 2019.
2. HAMAP as SPARQL tutorial. 2019. https://github.com/sib-swiss/HAMAP-SPARQL. Accessed 30 Nov 2019.
3. HAMAP website. https://hamap.expasy.org. Accessed 30 Nov 2019.
4. Lewin, H.A., et al. Earth BioGenome Project: Sequencing life for the future of life. *Proc Natl Acad Sci U S A* 2018;115(17):4325-4333.
5. Mukherjee, S., et al. 1,003 reference genomes of bacterial and archaeal isolates expand coverage of the tree of life. *Nat Biotechnol* 2017;35(7):676-683.
6. Paez-Espino, D., et al. Uncovering Earth's virome. Nature 2016;536(7617):425-430.
7. Thompson, L.R., et al. A communal catalogue reveals Earth's multiscale microbial diversity. *Nature* 2017;551(7681):457-463.
8. Tighe, S., et al. Genomic Methods and Microbiological Technologies for Profiling Novel and Extreme Environments for the Extreme Microbiome Project (XMP). *J Biomol Tech* 2017;28(1):31-39.
9. Meyer, F., et al. MG-RAST version 4-lessons learned from a decade of low-budget ultra-high-throughput metagenome analysis. *Brief Bioinform* 2017.
10. Overbeek, R., et al. The SEED and the Rapid Annotation of microbial genomes using Subsystems Technology (RAST). *Nucleic Acids Res* 2014;42(Database issue):D206-214.
11. Chen, I.A., et al. IMG/M: integrated genome and metagenome comparative data analysis system. *Nucleic Acids Res* 2017;45(D1):D507-D516.
12. Haft, D.H., et al. RefSeq: an update on prokaryotic genome annotation and curation. *Nucleic Acids Res*, Volume 46, Issue D1, 4 January 2018, Pages D851–D860,
13. Mitchell, A.L., et al. InterPro in 2019: improving coverage, classification and access to protein sequence annotations. *Nucleic Acids Res* 2019;47(D1):D351-D360.
14. Haft, D.H., et al. TIGRFAMs and Genome Properties in 2013. *Nucleic Acids Res* 2013;41(Database issue):D387-395.
15. Pedruzzi, I., et al. HAMAP in 2015: updates to the protein family classification and annotation system. *Nucleic Acids Res* 2015;43(Database issue):D1064-D1070.
16. HAMAP-Scan web service. https://hamap.expasy.org/hamap_scan.html. Accessed 30 Nov 2019.
17. SPARQL Query Language for RDF. 2013. https://www.w3.org/2001/sw/wiki/SPARQL. Accessed 30 Nov 2019.
18. Resource Description Framework (RDF). 2014. https://www.w3.org/RDF. Accessed 30 Nov 2019.
19. Bolleman, J.T., et al. FALDO: a semantic standard for describing the location of nucleotide and protein feature annotation. *J Biomed Semantics* 2016;7:39.
20. Zerbino, D.R., et al. *Ensembl* 2018. Nucleic Acids Res 2018;46(D1):D754-D761.
21. Kersey, P.J., et al. Ensembl Genomes 2018: an integrated omics infrastructure for non-vertebrate species. *Nucleic Acids Res* 2018;46(D1):D802-D808.
22. Ison, J., et al. EDAM: an ontology of bioinformatics operations, types of data and identifiers, topics and formats. *Bioinformatics* 2013;29(10):1325-1332.
23. Chibucos, M.C., et al. Standardized description of scientific evidence using the Evidence Ontology (ECO). *Database (Oxford)* 2014;2014.
24. Petersen, T.N., et al. SignalP 4.0: discriminating signal peptides from transmembrane regions. *Nat Methods* 2011;8(10):785-786.
25. Sonnhammer, E.L., von Heijne, G. and Krogh, A. A hidden Markov model for predicting transmembrane helices in protein sequences. *Proc Int Conf Intell Syst Mol Biol* 1998;6:175-182.
26. Schuepbach, T., et al. pfsearchV3: a code acceleration and heuristic to search PROSITE profiles. *Bioinformatics* 2013;29(9):1215-1217.
27. The Gene Ontology Consortium. The Gene Ontology Resource: 20 years and still GOing strong. *Nucleic Acids Res* 2019;47(D1):D330-D338.
28. McDonald, A.G., Boyce, S. and Tipton, K.F. ExplorEnz: the primary source of the IUBMB enzyme list. *Nucleic Acids Res* 2009;37(Database issue):D593-597.
29. Bairoch, A. The ENZYME database in 2000. *Nucleic Acids Res* 2000;28(1):304-305.
30. Lombardot, T., et al. Updates in Rhea: SPARQLing biochemical reaction data. *Nucleic Acids Res* 2018;47(D1):D596-D600.
31. Hastings, J., et al. ChEBI in 2016: Improved services and an expanding collection of metabolites. *Nucleic Acids Res* 2016;44(D1):D1214-1219.
32. PfTools v3. 2013. https://github.com/sib-swiss/pftools3. Accessed 30 Nov 2019.
33. XSLT stylesheet to convert InterProScan XML to RDF. 2019. https://github.com/sib-swiss/HAMAP-SPARQL/blob/master/src/main/xlst/interproToRdf.xslt. Accessed 30 Nov 2019.
34. HAMAP SPARQL server. 2019. https://hamap.expasy.org/sparql. Accessed 30 Nov 2019.
35. Fa, R., et al. Predicting human protein function with multitask deep neural networks. *PLoS One* 2018;13(6):e0198216.
36. Kulmanov, M., et al. DeepGO: predicting protein functions from sequence and interactions using a deep ontology-aware classifier. *Bioinformatics* 2018;34(4):660-668.
37. LOD4ML: Linked Open Data for Machine Learning. 2017. http://lod4ml.org. Accessed 30 Nov 2019.
38. R2RML: RDB to RDF Mapping Language. 2012. https://www.w3.org/TR/r2rml/. Accessed 30 Nov 2019.
39. Schmidt, M., Meier, M. and Lausen, G. Foundations of SPARQL query optimization. *Proceedings of the 13th International Conference on Database Theory* 2010:4-33.
40. The RNAcentral Consortium. RNAcentral: a comprehensive database of non-coding RNA sequences. *Nucleic Acids Res* 2017;45(D1):D128-D134.
41. Kalvari, I., et al. Rfam 13.0: shifting to a genome-centric resource for non-coding RNA families. *Nucleic Acids Res* 2018;46(D1):D335-D342.
42. Sigrist, C.J., et al. New and continuing developments at PROSITE. *Nucleic Acids Res* 2013;41(Database issue):D344-347.
43. The UniProt Consortium. UniProt: a worldwide hub of protein knowledge. *Nucleic Acids Res* 2019;47(D1):D506-D515.
44. Morgat, A., et al. Enzyme annotation in UniProtKB using Rhea. *Bioinformatics* 2019;btz817.
45. Faria, J.P., et al. Methods for automated genome-scale metabolic model reconstruction. *Biochem Soc Trans* 2018;46(4):931-936.
46. Moretti, S., et al. MetaNetX/MNXref–reconciliation of metabolites and biochemical reactions to bring together genome-scale metabolic networks. *Nucleic Acids Res* 2016;44(D1):D523-526.
47. Bolleman JT; de Castro E; Baratin D; Cuche B; Redaschi N; Bridge A: Supporting data for "HAMAP as SPARQL rules - A

portable annotation pipeline for genomes and proteomes" GigaScience Database. 2020. http://dx.doi.org/10.5524/100683

Click here to access/download
Supplementary Material
bolleman_supplement.tex

Click here to access/download
Supplementary Material
bolleman_supplement.pdf
