# Supplementary material for: HAMAP as SPARQL rules—A portable annotation pipeline for genomes and proteomes
Source: Gigascience. 2020 Feb 8;9(2):giaa003. doi: 10.1093/gigascience/giaa003 (PMC7007698; doi:10.1093/gigascience/giaa003)
Supplement: giaa003_Supplemental_File [file giaa003_supplemental_file.pdf]

1 Supplementary information

1.1 Supplement 1: Map position on template to target sequence using SPARQL 1.1 standard functions

The InterProScan software represents a sequence/signature alignment in the form of a string where an upper-case letter represents a matched position, a lower-case letter an inserted position and a dash ('-') a deleted position in the sequence with respect to the signature. This example illustrates how two alignment strings, one for a template/signature and the other for a target/signature alignment, can be used to map a sequence position from a template to a target sequence. This code is not expected to be typed by hand, but generated by tools as needed.

```
#-------------------------------------------------------------------------------
# Step 1: Map a given template sequence position to the signature.
#-------------------------------------------------------------------------------

BIND(87 AS ?templatePosition)

# Make a regular expression to find the position in the alignment string that
# corresponds to the template position (adjusted by the template sequence’s
# begin position in the alignment) that we need to map: The expression is bound
# to the start of the string and consists of repeats of a pattern of 0-n
# deletions (dashes) followed by 1 matched position or insertion (non-dash
# char).
BIND(
  CONCAT(’^(?:-*[^-]){’,
         STR((?templatePosition - ?templateAlignBeginPosition + 1)),
         ’}’)
  AS
  ?regexForSubstringToTemplatePosition)

# Count the number of chars present after the template position.
BIND(
  STRLEN(REPLACE(?templateAlign, ?regexForSubstringToTemplatePosition, ’’))
  AS
  ?lengthOfTemplateAlignAfterTemplatePosition)

# Extract the alignment substring up to the template position.
BIND(
  SUBSTR(?templateAlign,
         1,
         (STRLEN(?templateAlign)
         - ?lengthOfTemplateAlignAfterTemplatePosition
         ))
  AS
  ?substringToTemplatePosition)

# The position on the signature is the length of the above substring without the
# insertions (lower-case letters).
BIND(
  STRLEN(REPLACE(?substringToTemplatePosition, ’[a-z]’, ’’))
  AS
  ?signaturePosition)

#-------------------------------------------------------------------------------
# Step 2: Map the signature position to the target sequence.
#-------------------------------------------------------------------------------
```

```
# Make a regular expression to find the position in the alignment string that
# corresponds to the signature position that we need to map: The expression is
# bound to the start of the string and consists of repeats of a pattern of 0-n
# insertions (lower-case letters) followed by 1 matched position or deletion
# (upper-case letter or dash).
BIND(
  CONCAT('^(?:[a-z]*(?:[A-Z]|-)){',
         STR(?signaturePosition),
         '}')
  AS
  ?regexForSubstringToSignaturePosition)

# Count the number of chars present after the signature position.
BIND(
  STRLEN(REPLACE(?targetAlign, ?regexForSubstringToSignaturePosition, ''))
  AS
  ?lengthOfTargetAlignAfterSignaturePosition)

# Extract the alignment substring up to the signature position.
BIND(
  SUBSTR(?targetAlign,
         1,
         (STRLEN(?targetAlign)
         - ?lengthOfTargetAlignAfterSignaturePosition
         ))
  AS
  ?substringToSignaturePosition)

# The position on the target is the length of the above substring without the
# deletions (dashes), adjusted by the target sequence's begin position in the
# alignment.
BIND(
  (STRLEN(REPLACE(?substringToSignaturePosition, '[-]', '')) + ?targetAlignBeginPosition - 1)
  AS
  ?signaturePosition)
```

1.2 Supplement 2: Java Apache Jena ARQ custom function

```java
package org.expasy.hamap.tools.sparql.jena.functions;

import java.util.List;

import org.apache.jena.query.QueryBuildException;
import org.apache.jena.sparql.expr.ExprEvalException;
import org.apache.jena.sparql.expr.ExprList;
import org.apache.jena.sparql.expr.NodeValue;
import org.apache.jena.sparql.function.FunctionBase;
import org.apache.jena.sparql.util.FmtUtils;


/**
 * @see https://jena.apache.org/documentation/query/writing_functions.html
 *
 */
public class PositionalFeatureShifter
    extends FunctionBase
{

  private static final int NUMBER_EXPECTED_ARGUMENTS = 5;

  public PositionalFeatureShifter()
  {
    super();
  }

  @Override
  public NodeValue exec(List<NodeValue> args)
  {
    if (args.size() != 5)
      throw new ExprEvalException("Incorrect number of arguments");
    return exec(args.get(0), args.get(1), args.get(2), args.get(3), args.get(4));
  }

  public NodeValue exec(NodeValue alignStringSignatureTemplate, NodeValue signatureTemplateStart,
      NodeValue positionToMap,
      NodeValue alignStringSignatureTarget, NodeValue signatureTargetStart)
  {
    if (!alignStringSignatureTemplate.isString())
      throw new ExprEvalException("Not a String: "
        + FmtUtils.stringForNode(alignStringSignatureTemplate.asNode()));
    else if (!alignStringSignatureTarget.isString())
      throw new ExprEvalException("Not a String: "
```

```
      + FmtUtils.stringForNode(alignStringSignatureTarget.asNode()));
  else if (!positionToMap.isNumber())
    throw new ExprEvalException("Not a number: "
      + FmtUtils.stringForNode(positionToMap.asNode()));
  else if (!signatureTemplateStart.isNumber())
    throw new ExprEvalException("Not a number: "
      + FmtUtils.stringForNode(signatureTemplateStart.asNode()));
  final String templateAlign = alignStringSignatureTemplate.asUnquotedString();
  final String targetAlign = alignStringSignatureTarget.asUnquotedString();

  // Step 1: Count the non-insertion chars in the 'template to signature' mapping.
  int templatePositionInt = positionToMap.getInteger().intValue()
      - (signatureTemplateStart.getInteger().intValue()) + 1;
  final int step1 = fromTemplateToSignature(templatePositionInt, templateAlign) ;

  // Step 2: Count the non-deletion chars in the 'signature to target' mapping,
  // using the signature position calculated in step1.
  final int step2 = fromSignatureToTarget(step1, targetAlign);
  final int mappedPosition = step2 + signatureTargetStart.getInteger().intValue() - 1;

  return NodeValue.makeInteger(mappedPosition);
}

static final int fromTemplateToSignature(final int pos, final String align)
{
  int nonDeletionCounter = 0;
  int nonInsertionCounter = 0;
  for (int i = 0; i < align.length(); i++)
  {
    if (align.charAt(i) == '-' || Character.isUpperCase(align.charAt(i)))
    {
      nonInsertionCounter++;
    }
    if (align.charAt(i) != '-')
      nonDeletionCounter++;
    if (nonDeletionCounter == pos)
      return nonInsertionCounter;
  }
  throw new ExprEvalException("");
}

static final int fromSignatureToTarget(final int pos, final String align)
{
  int nonDeletionCounter = 0;
  int nonInsertionCounter = 0;
  for (int i = 0; i < align.length(); i++)
```

```
    {
      if (align.charAt(i) == '-' || Character.isUpperCase(align.charAt(i)))
      {
        nonInsertionCounter++;
      }
      if (align.charAt(i) != '-')
        nonDeletionCounter++;
      if (nonInsertionCounter == pos)
        return nonDeletionCounter;
    }
    throw new ExprEvalException("");
  }

  @Override
  public void checkBuild(String uri, ExprList args)
  {
    if (args.size() != NUMBER_EXPECTED_ARGUMENTS)
      throw new QueryBuildException(
          "Function '" + this.getClass() + "' takes " + NUMBER_EXPECTED_ARGUMENTS + " arguments");
  }
}
```

1.3 Supplement 3: XSLT to convert InterProScan XML output to minimal RDF for HAMAP

This XSLT stylesheet transforms the XML result file of a local InterProScan run (with the -dp option) into the minimal set of RDF triples required by HAMAP SPARQL rules.

```
<?xml version="1.0"?>
<xsl:stylesheet version="1.0"
    xmlns:xsl="http://www.w3.org/1999/XSL/Transform"
    xmlns:in="http://www.ebi.ac.uk/interpro/resources/schemas/interproscan5"
    xmlns:rdf="http://www.w3.org/1999/02/22-rdf-syntax-ns#"
    xmlns:faldo="http://biohackathon.org/resource/faldo#"
    xmlns:fn="http://www.w3.org/2005/xpath-functions"
    xmlns:up="http://purl.uniprot.org/core/"
    xmlns:rdfs="http://www.w3.org/2000/01/rdf-schema#">
  <xsl:output method="text"/>

  <xsl:template match="/">
PREFIX up:&lt;http://purl.uniprot.org/core/&gt;
PREFIX rdf:&lt;http://www.w3.org/1999/02/22-rdf-syntax-ns#&gt;
PREFIX rdfs:&lt;http://www.w3.org/2000/01/rdf-schema#&gt;
PREFIX faldo:&lt;http://biohackathon.org/resource/faldo#&gt;
PREFIX signature:&lt;http://purl.uniprot.org/hamap/&gt;
PREFIX edam: &lt;http://edamontology.org/&gt;

    <xsl:apply-templates />
  </xsl:template>

  <xsl:template match="in:protein-matches">
    <xsl:for-each select="in:protein">
      <xsl:variable name="sequenceid" select="translate(in:xref/@id, '|', '%7C')"/>
      <xsl:variable name="sequencemd5" select="translate(in:sequence/@md5,'acbdef','ABCDEF')"/>

    <xsl:for-each select="in:matches/in:profilescan-match">
      <xsl:variable name="signatureid" select="in:signature/@ac"/>
      <xsl:variable name="start" select="in:locations/in:profilescan-location/@start"/>
      <xsl:variable name="end" select="in:locations/in:profilescan-location/@end"/>
&lt;<xsl:value-of select="$sequenceid"/>&gt;
  up:sequence &lt;<xsl:value-of select="$sequenceid"/>-sequence&gt; ;
  rdfs:seeAlso signature:<xsl:value-of select="$signatureid"/> .
  </xsl:for-each>
&lt;<xsl:value-of select="$sequenceid"/>-sequence&gt;
  rdf:hasValue "<xsl:value-of select="in:sequence"/>" .

    <xsl:for-each select="in:matches/in:profilescan-match">
      <xsl:variable name="signatureid" select="in:signature/@ac"/>
```

```
      <xsl:variable name="start" select="in:locations/in:profilescan-location/@start"/>
      <xsl:variable name="alignment" select="in:locations/in:profilescan-location/in:alignment/text()"/>
      <xsl:variable name="end" select="in:locations/in:profilescan-location/@end"/>
[] a edam:data_0869 ;
  <xsl:if test="$alignment != 'Not available'">
  rdf:value "<xsl:value-of select="$alignment" />" ;
  </xsl:if>
  edam:is_output_of [
    a edam:operation_0300 ;
    edam:has_input signature:<xsl:value-of select="$signatureid"/>
  ] ;
  faldo:begin [ faldo:position <xsl:value-of select="$start" />] ;
  faldo:end   [ faldo:position <xsl:value-of select="$end" /> ] .
      </xsl:for-each>
    </xsl:for-each>
  </xsl:template>
</xsl:stylesheet>
```
