# Supplementary material for: HAMAP as SPARQL rules—A portable annotation pipeline for genomes and proteomes
Source: Gigascience. 2020 Feb 8;9(2):giaa003. doi: 10.1093/gigascience/giaa003 (PMC7007698; doi:10.1093/gigascience/giaa003)
Supplement: giaa003_Response_to_Reviewer_Comments_Original_Submission [file giaa003_response_to_reviewer_comments_original_submission.pdf]

# Author's Response To Reviewer Comments

Close

Dear Reviewers, Curators, Editor,

Thank you for your kind reviews and patience.

===========

COMMENT: a key requirement for GigaScience is usability, and more detailed documentation, tutorial and example should be provided.
ANSWER: We have published documentation and a tutorial at https://github.com/sib-swiss/HAMAP-SPARQL

COMMENT: please register any new software application in the bio.tools and SciCrunch.org databases to receive RRID (Research Resource Identification Initiative ID) and biotoolsID identifiers, and include these in your manuscript. This will facilitate tracking, reproducibility and re-use of your tool.

ANSWER: HAMAP is already registered with RRID:SCR_007701.

Reviewer #1:
============

COMMENT: No citation or other information is provided to support this claim on the level of detail and quality of the annotations that HAMAP rules produce. Either a citation to work showing this should be provided or the sentence altered.

ANSWER: The process of UniProtKB/Swiss-Prot curation and HAMAP rule curation are tightly coupled. HAMAP rules are created by the same Swiss-Prot curators that curate the template entries. These curators then extract the relevant annotations into HAMAP rules. The HAMAP rules are also checked against the content of matching UniProtKB/Swiss-Prot entries at each release - to ensure that the rules remain consistent with the current contents of UniProtKB/Swiss-Prot. So we can reasonably say that the HAMAP system is of the same detail and quality as UniProtKB/Swiss-Prot. We have added the missing citation that describes this in more detail. Interested users can search for HAMAP annotation at uniprot.org using this url: https://www.uniprot.org/uniprot/?query=reviewed:yes+source:hamap.

Reviewer #2:
============

COMMENT: The package (ftp://ftp.expasy.org/databases/hamap/hamap_sparql.tar.gz) only contains several data files, without documentation
ANSWER: We have added a README file to the package that describes where to find the documentation. This points to https://github.com/sib-swiss/HAMAP-SPARQL.

COMMENT: It will be more convincing if rules from another popular annotation pipeline cited in the paper can be made into SPARQL query. This can be included either in the paper, supplementary materials, or an example packed with the software.

ANSWER: One of the most popular annotation tools is InterPro2GO (described in the already cited InterPro paper). We show how to

translate these simple association rules in
https://github.com/sib-swiss/HAMAP-SPARQL/blob/master/INTERPRO2GO.md.

COMMENT: Supplementary document is in Latex format, which is not very convenient to read. I suggest convert it to PDF format.
ANSWER: Our apologies, we had expected the gigascience pipeline to have done this for us, We have also published the code contained in the supplement at
https://github.com/sib-swiss/HAMAP-SPARQL

COMMENT: Documentation of the tool is very limited. More detailed documentation, tutorial and example should be provided.
ANSWER: We have published documentation and a tutorial at
https://github.com/sib-swiss/HAMAP-SPARQL

COMMENT: Citations were not correctly ordered.
ANSWER: We have reordered the citations according to the guidelines for authors.

Curators:
============

To make it possible to reproduce the query described in Figure 6 we have the data required in nquads format in a compressed form. We would like to upload this to gigadb.

ALL:
===========

We also rephrased the section on performance to apply to a more common to understand task, annotating a bacterial genome instead of a whole Swiss-Prot release.

With sincere regards,
The authors

Close
