# Supplementary material for: HAMAP as SPARQL rules—A portable annotation pipeline for genomes and proteomes
Source: Gigascience. 2020 Feb 8;9(2):giaa003. doi: 10.1093/gigascience/giaa003 (PMC7007698; doi:10.1093/gigascience/giaa003)
Supplement: giaa003_Response_to_Reviewer_Comments_Revision_1 [file giaa003_response_to_reviewer_comments_revision_1.pdf]

Author's Response To Reviewer Comments

Close

Dear Editors,

We have included the DOI as requested. I also made a single change to the first reference which was pointing to a compressed file on the HAMAP ftp site. This was changed to a directory as the contents of the compressed file are now available there directly.

Regards,
Jerven

Close
