# Supplementary material for: HAMAP as SPARQL rules—A portable annotation pipeline for genomes and proteomes
Source: Gigascience. 2020 Feb 8;9(2):giaa003. doi: 10.1093/gigascience/giaa003 (PMC7007698; doi:10.1093/gigascience/giaa003)
Supplement: giaa003_Reviewer_1_Report_Original_Submission — Mark Wass -- 8/12/2019 Reviewed [file giaa003_reviewer_1_report_original_submission.pdf]

Reviewer Report

Title: HAMAP as SPARQL rules - A portable annotation pipeline for genomes and proteomes

Version: Original Submission **Date:** 8/12/2019

Reviewer name: Mark Wass

Reviewer Comments to Author:

This manuscript details the provision of HAMAP rules for functional annotation of proteins using SPARQL. The aim is to make it simple for researchers to annotate a set of sequences or a genome with HAMAP rules. This is a resource that many are likely to find useful. The manuscript is clearly written and detailed.

I have just one minor comment. The introduction states the following "HAMAP classifies and annotates protein sequences using a collection of expert-curated protein family signatures and annotation rules. These rules annotate family members to the same level of detail and quality as expert curated UniProtKB/Swiss-Prot records, combining family membership and residue dependencies to ensure a high degree of specificity."

No citation or other information is provided to support this claim on the level of detail and quality of the annotations that HAMAP rules produce. Either a citation to work showing this should be provided or the sentence altered.

Methods

Are the methods appropriate to the aims of the study, are they well described, and are necessary controls included? Choose an item.

Conclusions

Are the conclusions adequately supported by the data shown? Choose an item.

Reporting Standards

Does the manuscript adhere to the journal's guidelines on minimum standards of reporting? Choose an item.

Choose an item.

Statistics

Are you able to assess all statistics in the manuscript, including the appropriateness of statistical tests used? Choose an item.

Quality of Written English

Please indicate the quality of language in the manuscript: Choose an item.

Declaration of Competing Interests

Please complete a declaration of competing interests, considering the following questions:

- Have you in the past five years received reimbursements, fees, funding, or salary from an organisation that may in any way gain or lose financially from the publication of this manuscript, either now or in the future?
- Do you hold any stocks or shares in an organisation that may in any way gain or lose financially from the publication of this manuscript, either now or in the future?
- Do you hold or are you currently applying for any patents relating to the content of the manuscript?
- Have you received reimbursements, fees, funding, or salary from an organization that holds or has applied for patents relating to the content of the manuscript?
- Do you have any other financial competing interests?
- Do you have any non-financial competing interests in relation to this paper?

If you can answer no to all of the above, write 'I declare that I have no competing interests' below. If your reply is yes to any, please give details below.

I declare that I have no competing interests

I agree to the open peer review policy of the journal. I understand that my name will be included on my report to the authors and, if the manuscript is accepted for publication, my named report including any attachments I upload will be posted on the website along with the authors' responses. I agree for my report to be made available under an Open Access Creative Commons CC-BY license (http://creativecommons.org/licenses/by/4.0/). I understand that any comments which I do not wish to be included in my named report can be included as confidential comments to the editors, which will not be published.

Choose an item.

To further support our reviewers, we have joined with Publons, where you can gain additional credit to further highlight your hard work (see: https://publons.com/journal/530/gigascience). On publication of this paper, your review will be automatically added to Publons, you can then choose whether or not to claim your Publons credit. I understand this statement.

Yes Choose an item.
